# Overexpression of *MusaVicilin* gene for disease resistance in banana

**DOI:** 10.3389/fpls.2026.1737976

**Published:** 2026-03-23

**Authors:** Sarah Wanjiku Macharia, Jaindra Nath Tripathi, Valentine Otang Ntui, Samwel Muiruri Kariuki, Leena Tripathi

**Affiliations:** 1 International Institute of Tropical Agriculture (IITA), Nairobi, Kenya; 2 Department of Plant Sciences, Kenyatta University, Nairobi, Kenya

**Keywords:** Banana Xanthomonas wilt, constitutive overexpression, genetic modification, *MusaVicilin*, *Xanthomonas vasicola* pv. *musacearum*

## Abstract

Banana Xanthomonas wilt (BXW) disease, caused by *Xanthomonas vasicola* pv. *musacearum*, is a major constraint to banana production in East and Central Africa. All cultivated banana varieties are susceptible, with the wild progenitor *Musa balbisiana* being the only known source of complete resistance. Limitations in classical breeding have prompted the exploration of molecular genetic tools, such as genetic modification, to develop resistant cultivars. Comparative transcriptomic analyses revealed a five-fold upregulation of *MusaVicilin* gene in *M. balbisiana* (BB genome) compared to the BXW- susceptible ‘Pisang Awak’ at early infection stage with the pathogen, suggesting its role in defense. This study investigated whether constitutive overexpression of the *MusaVicilin* gene cloned from *M. balbisiana* could enhance resistance to BXW in the susceptible ‘Sukali Ndiizi’ cultivar (AAB genome). Transgenic lines were developed overexpressing the *MusaVicilin* gene under the control of the constitutive CaMV 35S promoter. *Agrobacterium*-mediated transformation of embryogenic cell suspension of ‘Sukali Ndiizi’ generated 52 independent transgenic lines. These transgenic lines were confirmed via PCR and Southern blot analysis. The transgenic lines showed reduced disease severity and significantly lower disease severity index compared with non-transgenic controls. *MusaVicilin* overexpression (∼400 - 1300 folds) showed moderate correlation with disease resistance (r=0.68, p<0.01), with transgenic line (S2) demonstrating complete resistance under greenhouse conditions. These results suggest that the overexpression of *MusaVicilin* can confer enhanced resistance to BXW and highlight its potential as a candidate gene for genetic engineering resistance to BXW in susceptible cultivars. Moreover, *MusaVicilin* could serve as a valuable component in gene stacking strategies aimed at developing durable, disease-resistant banana varieties.

## 1 Introduction

Bananas (*Musa* spp.) hold critical importance in global food security, feeding over 400 million people worldwide (Tripathi et al., 2024). Cultivated in more than 140 countries in the subtropics and tropics, bananas have a global annual production of approximately 183.6 million tonnes, with India as the leading producer approximately 36.6 million tonnes (FAOSTAT, 2023). Bananas are among the most widely consumed fruits globally, with consumption projected to reach 90 million metric tonnes by 2026 (ReportLinker (2022-2026)). In East Africa, bananas contribute significantly to daily per-person calorie intake, ranging from 30% to 60%, with Uganda having the highest consumption rates (Abele et al., 2007). Cultivated bananas are allopolyploids or autopolyploids of *Musa acuminata* (AA genome) and *Musa balbisiana* (BB genome). The diversity of banana cultivars is estimated to be 300 to 1,200, belonging to various genomic groups such as AA, BB, AB, AAA, AAB, ABB, AAAA, AAAB, AABB, and ABBB (Šimoníková et al., 2020).

Banana production in East and Central Africa faces severe challenges due to Banana Xanthomonas Wilt (BXW), a systemic bacterial disease caused by *Xanthomonas vasicola* pv. *musacearum (Xvm)* (Studholme et al., 2020); previously known as *X. campestris* pv. *musacearum (Xcm).* This devastating disease affects all cultivated bananas and can lead to yield losses of up to 100% (Tripathi et al., 2009). BXW has resulted in the destruction of entire plantations in affected regions, compelling farmers to seek infection-free planting sites (Tripathi et al., 2022). The economic toll of BXW is estimated to range from $2 to $8 billion over the course of a decade (Abele and Pillay, 2007). Transmission of *Xvm* occurs through various means, including insect vectors, contaminated tools, soil-borne bacterial inoculum, and infected planting material (Tripathi et al., 2009).

In the absence of BXW-resistant cultivated varieties, a comprehensive approach is required for effective management of BXW disease. Strategies such as rigorous phytosanitary measures, careful decapitation of male buds (Tripathi et al., 2014b), and strict adherence to hygiene protocols (Tripathi et al., 2009) are crucial for preventing disease transmission. However, such strategies are inherently limited: their effectiveness depends heavily on farmer compliance, they are labor-intensive, and they cannot fully eliminate the pathogen, which may also adapt to management practices (Biruma et al., 2007). This emphasizes the need for the development of improved banana varieties with enhanced resistance to BXW, in addition to continued research into comprehensive control strategies (Tripathi et al., 2010, 2014b; Nakato et al., 2019).

While all cultivated banana varieties are vulnerable to BXW disease, the wild progenitor *M. balbisiana* exhibits complete resistance (Tripathi et al., 2009, 2019). Moreover, Nakato et al. (2019) noted significant *Xvm* tolerance in the AA genome of *Musa acuminata* subsp. zebrina, implying the existence of tolerant traits within the current banana germplasm. Transferring the disease resistance trait from wild-type banana *M. balbisiana* to farmer-preferred cultivars through conventional breeding is a lengthy and challenging process due to the sterility (Namukwaya et al., 2012) of most cultivars coupled with polyploidy and the long generation times. Additionally, the presence of banana streak virus (BSV) sequences in the B genome limits the use of *M. balbisiana* in conventional breeding (Iskra-Caruana et al., 2014). These barriers highlight the urgency for innovative biotechnological interventions such as genetic engineering to revolutionize banana breeding, ultimately fostering the development of resilient and improved banana varieties.

Genetic engineering has shown promising progress by incorporating defense genes such as *Hrap, Pflp, Xa21*, *ES-Pflp*, and *AtEFR* into banana plants (Tripathi et al., 2010, 2014a, 2019; Wanjiku et al., 2021; Adero et al., 2023). However, the continual evolution of new pathogenic strains and the adaptability of *Xvm* pose ongoing challenges to disease eradication efforts. To overcome these obstacles, it is crucial to explore the genetic potential of the BXW-resistant wild banana varieties (Heslop-Harrison and Schwarzacher, 2007; Tripathi et al., 2019; Namukwaya et al., 2012; Iskra-Caruana et al., 2014).

Comparative transcriptomics between the BXW-susceptible banana cultivar ‘Pisang Awak’ and BXW-resistant *M. balbisiana* during early *Xvm* infection identified numerous defense-related genes. Notably, *MusaVicilin* was strongly upregulated in *M. balbisiana* but downregulated in the susceptible cultivar, highlighting the rationale for overexpressing *MusaVicilin* in susceptible banana cultivars to enhance resistance (Tripathi et al., 2019).

Vicilins are 7S globulins with molecular weights typically between 200 and 400 kDa and exhibit a complex range of functions. They are predominant in legumes and are primarily known as seed storage proteins. They also serve as precursors for antimicrobial peptides (AMPs) (Christelová et al., 2011), specifically vicilin-like AMPs, that are released through proteolytic cleavage during seed development or in response to pathogen attack. During this process, vicilin proteins are cleaved at precise sites by vacuolar processing enzymes, or defense-related proteases, releasing smaller bioactive vicilin-like peptides that exhibit antimicrobial properties (Lay and Anderson, 2005; Nakato et al., 2019). These AMPs form an integral part of the plant’s innate immune system, combating bacteria, fungi, viruses, yeast, and pests (Broekaert et al., 1997; Gomes et al., 1998; Ryan et al., 2007; Marcus et al., 2008; Tam et al., 2015; Gil-Salido et al., 2024).

Vicilin-like peptides have demonstrated significant antimicrobial properties, contributing to plant defense against various pathogens. Vicilin-like proteins from *Capsicum baccatum* L., narrow-leafed lupin, and *Clitoria fairchildiana* exhibited antifungal and insecticidal properties (Vieira Bard et al., 2014; Bertonceli et al., 2022). Cysteine-rich vicilin peptides from *Centrosema virginianum* improved fungal resistance in transgenic tobacco (Xie et al., 2016), while antimicrobial peptides derived from the N-terminus region of vicilin proteins in *Macadamia integrifolia* and *Theobroma cacao* (Marcus et al., 1999), also displayed potent antifungal activity against *Fusarium oxysporum* and *Aschochyta rabiae.* Additionally (Almeida et al., 2022), revealed antibacterial activity in hydrolysates of cowpea vicilin. Further, binding to chitin, vicilins from cowpea, and *Enterolobium contortisiliquum* seeds interfered with insect larval development and prevented fungal germination (Sales et al., 2001; Moura et al., 2007). These findings collectively highlight the broad-spectrum defensive capabilities of vicilin-derived antimicrobial peptides against bacterial, fungal, and insect pests across diverse plant species.

Building on these insights and previous transcriptome analysis, *MusaVicilin* was identified as a potential candidate gene associated with resistance to BXW. To investigate its functional role, *MusaVicilin* was constitutively overexpressed in the BXW-susceptible cultivar ‘Sukali Ndiizi’. Transgenic lines overexpressing *MusaVicilin* were evaluated under greenhouse conditions following challenge with *Xvm*. The transgenic lines exhibited significantly enhanced resistance compared with non-transgenic controls, confirming that *MusaVicilin* is associated with enhanced resistance to BXW. These findings highlight *MusaVicilin* gene as a promising target for developing BXW-resistant banana cultivars.

## 2 Materials and methods

### 2.1 Bioinformatics analysis and identification of *MusaVicilin* conserved domains

To identify the known conserved vicilin domains in MusaVicilin relative to well characterized legumes, protein sequences were retrieved from the National Center for Biotechnology Information (NCBI) protein database based on a list curated from literature search. The sequences included broad bean (*Vicia faba* – accession CAI8588729.1) (Bailey and Boulter, 1972), pea (*Pisum sativum* – accession XP_050906640.1) (Gatehouse et al., 1981; I'Anson et al., 1988), common bean (*Phaseolus vulgaris –* accession XP_068467458.1) (Bollini and Chrispeels, 1978), eggplant (*Solanum melongena* – accession ABU45177.1) (Jain et al., 2016), Soybean (*Glycine max* – accession XP_003537573.1) (Doyle et al., 1986), Macademia (*Macademia integrifolia* – accession AAD54245.1) (Kabasser et al., 2022) and one monocot rice (Oryza sativa – accession ABF94466). The MusaVicilin protein sequence (accession XP_065008984.1) was obtained from the banana genome database (https://banana-genome-hub.southgreen.fr/). The protein IDs were used to query the NCBI Conserved Domain Database (CDD) through the batch-CD search tool using default search parameters to establish presence and location of conserved domains. The resulting protein domains were presented schematically using Domain Graph (DOG 2.0) version 2.0 (Ren et al., 2009).

### 2.2 Phylogenetic analysis of MusaVicilin

To obtain vicilin sequences for use in phylogenetic analysis of MusaVicilin, a protein BLAST (BLASTp) using the MusaVicilin to query legume-specific sequences in the NCBI was done. The resulting sequences were downloaded in FASTA format. Multiple sequence alignment was performed on the sequences using MAFFT version 7 (Katoh and Standley, 2013), executed within the WSL Linux environment. The L-INS-i algorithm was selected to maximize alignment accuracy for sequences with potential local homology and variable domain architecture. Default gap opening and extension penalties were used. Problematic sequences including those with redundancy or unable to meet trimming criteria were completely excluded in the final alignment. The final analysis had 92 sequences, ninety from the leguminosae family (*Fabaceae*) and two monocots (banana and rice). The best-fitting amino acid substitution model was selected using ModelFinder (Kalyaanamoorthy et al., 2017). Branch support was assessed using the ultrafast bootstrap approximation with 1,000 replicates (Hoang et al., 2018). Phylogenetic tree was visualized and annotated using the Interactive Tree of Life (iTOL) web server, version 5 (Letunic and Bork, 2021). All strongly supported clades were highlighted with unique colour to distinguish them from others of different taxa. Branch lengths were displayed proportional to amino acid differences and similarities per site, and font sizes in the label were adjusted to improve readability.

### 2.3 Plant material

Embryogenic cell suspension (ECS) of banana cultivar ‘Sukali Ndiizi’ used for transformation was generated from immature male flowers (Tripathi et al., 2015) and maintained at 28 ± 2 °C on a rotary shaker at 95 rpm in the dark.

### 2.4 *MusaVicilin* gene construct design

The *MusaVicilin* gene sequences were downloaded from banana genome B (*M. balbisiana*), gene ID: Vicilin ITC1587_Bchr3_P08153 (Mba01_g02390.1) from the Banana Genome Hub (D’Hont et al., 2012). PCR primers (Mb_Vicilin_F: AGAGCGAGCGAGAGAGAGAA and Mb_Vicilin_R: CCTCTTCCCTTCACTCTGT) were designed to isolate the full-length gene (1664 bp) from *M. balbisiana* genomic DNA. PCR amplification was done using Hotstar Taq DNA polymerase (QIAGEN, Germany). After amplification, the PCR product was resolved in 1% agarose gel stained with GelRed (Biotium, San Francisco, USA), a fluorescent nucleic acid stain. The PCR product was purified using the PCR purification kit (QIAGEN, Hilden, Germany), according to the manufacturers’ instructions) and cloned into the Gateway entry vector, pCR8/GW/TOPO (Invitrogen, Carlsbad, CA, USA). The ligated product was transformed into *DH5α E. coli* competent cells and selected on LB medium containing spectinomycin (100 mg/l). Ten colonies were selected, and plasmid DNA was extracted. It was initially verified by PCR and later by Sanger sequencing using M13 primers. The transformants containing the correct *M. balbisiana* gene sequences were subcloned in the sense orientation between the attR1 and attR2 recombination sites in the binary vector pMDC32 by LR Clonase™ (Invitrogen, Carlsbad, CA, USA) recombination reaction to yield pMDC32*_MbVicilin*. The plasmid was transformed into *DH5α E. coli* competent cells and selected on LB medium containing kanamycin (50 mg/l). Ten independent colonies were selected and verified by digestion with *Eco*RI. The colony with the correct insert was introduced into the highly virulent *Agrobacterium tumefaciens* strain EHA105 by electroporation. Ten colonies were selected, and colony PCR was performed to check integration of the plasmid into the *Agrobacterium*. The pMDC32_*MbVicilin* binary vector containing *hygromycin phosphotransferase (hpt)* as the selection marker gene, and the *MusaVicilin* gene driven by the cauliflower mosaic virus (CaMV) 35S promoter was used for plant transformation.

### 2.5 Generation of transgenic plants

Transgenic lines overexpressing *MusaVicilin* were generated by delivering the pMDC32-35S::*MbVicilin* plasmid into the ECS of ‘Sukali Ndiizi’ by *Agrobacterium*-mediated transformation as described previously by Tripathi et al. (2015). The *Agrobacterium*-infected ECS were regenerated on a selective medium supplemented with 12.5 mg/l hygromycin following the protocol described by Tripathi et al. (2015). All regenerated putative transgenic lines were maintained and multiplied on proliferation medium at 28 ± 2 °C under a 16/8 h light/dark photoperiod with fluorescent lighting. The putative transgenic lines were validated for the presence of the *hpt* gene by PCR. Rooted plants were transferred to soil in the greenhouse for BXW screening.

### 2.6 PCR analysis of transgenic lines

Genomic DNA was extracted from freshly collected leaf samples of the putatively transgenic lines and non-transgenic control (NTC) plants using the cetyltrimethylammonium bromide (CTAB) method (Stewart and Via, 1993). All 52 hygromycin-resistant lines were assessed for transgenesis by targeting amplification of 415 bp long region of the *hpt* gene using the *hpt* specific primers (forward 5′ GATGTTGGCGACCTCGT 3′ and reverse 5′ GTGTCACGTTGCAAGACCTG 3′). The PCR reaction set up in a 25-μL reaction volume contained 2.5 μL 10X PCR buffer, 0.3 μL 10 mM dNTPs, 1 μL of 10 μM reverse and forward primers, 2 μL genomic DNA (100 ng/μL), 0.2 μL Taq DNA polymerase of 5 units/μL (Qiagen, Germany), and 18 μL nuclease-free water. BIO-RAD T100 PCR conditions: 95 °C/5 min; 32 cycles (94 °C/30 s, 56 °C/30 s, 72 °C/1 min); 72 °C/7 min. Genomic DNA from NTC plants was used as a negative control, and the pMDC32_*MbVicilin* plasmid DNA was used as a positive control. PCR products visualized on a 1% agarose gel (GelRed staining).

### 2.7 Plant growth analysis

Growth parameters were assessed for all 52 PCR-positive transgenic lines compared to NTC plants in the greenhouse. Three biological replicates of well-rooted plants for each line were acclimatized for 30 days in a humidity chamber, then grown in the greenhouse for 90 days at 25–30˚C. Growth parameters (height, pseudostem girth, functional leaves, and middle leaf dimensions), recorded at 90 days and total leaf area calculated (Kumar et al., 2002);


Total leaf area =0.8×L×W×N

In which L = length of the middle leaf, W = width of the middle leaf, and N = total number of leaves in the plant.

### 2.8 Disease evaluation under greenhouse conditions

Three biological replicates of all 52 transgenic lines and NTC plants were evaluated against *Xvm* resistance under greenhouse conditions (Tripathi et al., 2010, 2014a). Plants were arranged in a completely randomized design. The Ugandan *Xvm* strain (obtained from collection at IITA-Nairobi) was cultured in YPGA medium (48 h at 28 °C) (Mwangi et al., 2007), pelleted (4000 rpm, 15 min), resuspended to an OD_600nm_ =1. The freshly prepared bacterial culture was inoculated (100 μL) using an insulin syringe into second functional leaf of 90-days-old plants. Appearance of symptoms, including leaf drooping or wilting, necrosis, and complete wilting of the plantlets were monitored daily over a 60-day post-inoculation (dpi) period and resistance calculated compared to NTC plants (Tripathi et al., 2014a).


Resistance (%)=(Reduction in wilting of transgenic line/number of leaves wilted in the control plant)×100

Disease severity was recorded on a scale of 0–5 (0—no symptoms, 1—only the leaf inoculated wilted, 2—two to three leaves wilted, 3—four to five leaves wilted, 4—all the leaves wilted but the plant still alive, 5—whole plant died) (Tripathi et al., 2025).

A disease severity index (DSI) was calculated for all the transgenic lines along with NTC.


DSI (%) = ∑ (Disease severity scale) X no. of plants in each scale)/(total number of plants) × (maximal disease severity scale)×100

### 2.9 Gene expression analysis

A subset of 11 transgenic lines overexpressing *MusaVicilin* and NTC plants were selected based on their disease evaluation results in the greenhouse. To determine the relative gene expression of these lines compared to the control NTC plant, total RNA was extracted from the leaves of two-week-old *in vitro* back up plants of lines screened using the Qiagen Plant RNeasy Kit, adhering to the manufacturer’s instructions. The RNA quality and concentration were determined using NanoDrop 2000 (Thermo Fisher Scientific). Subsequently, 1 μg of total RNA was converted to cDNA using the LunaScript^®^ RT SuperMix cDNA Synthesis Kit (New England BioLabs, E3010L) as outlined in the user’s manual. The resulting cDNA was diluted ten-fold, and 5 μl was used as a template for qRT-PCR with primers (*MusaVicilin* forward 5′ TCAGGGCGAGTCGATAATA 3′ and *MusaVicilin* reverse 5′ CTCTTCTTGGCTTCCTCTTC) and SYBR Green Master Mix (Applied Biosystems) on a QuantStudio5 real-time PCR system (Applied Biosystems, Thermo Fisher Scientific). The Musa 25S forward primer 5′ ACATTGTCAGGTGGGGAGTT 3′ and Musa 25S reverse primer 5′ CCTTTTG TTCCACACGAGATT 3′ served as the internal control (Tripathi et al., 2025). A non-template control, containing nuclease-free water, was also included in the analysis. Each sample consisted of three technical replicates. The reaction volume was 20 μl, consisting of 10 μl of 5X Luna Universal qPCR Master Mix, 0.2 μl of 10 μM forward and reverse primers, 5 μl cDNA, and 4.6 μl nuclease-free water. Real-time PCR conditions were as follows: initial denaturation at 95 °C for 5 min, followed by 40 cycles of denaturation at 94 °C for 30 s, annealing at 60 °C for 30 s, and extension at 72 °C for 1 amin. The relative gene expression levels were determined using the 2−ΔCt method (Livak and Schmittgen, 2001).

### 2.10 Southern blot analysis

Southern blot analysis was performed on the *MusaVicilin* overexpression lines as per the method described by Tripathi et al. (2014b). Briefly, 10 μg of genomic DNA from each sample was digested with the restriction enzyme *Hind*III at 37^0^ C for 12 h and separated on 0.8% agarose gel at 50 V for 5h. The plasmid pMDC32-35S::*MbVicilin* and genomic DNA from a NTC plant were used as positive and negative controls, respectively. The gel, stained with GelRed (Biotium, San Francisco, USA), was viewed under ultraviolet light (using Syngene™ Ingenius 3 gel documentation system) to confirm the digestion. The restricted DNA was denatured, then blotted onto a positively charged membrane (Roche Diagnostics, West Sussex, UK) and fixed by cross-linking in ultraviolet light (UV 500 Crosslinker, Amersham Biosciences). The membrane was then hybridized with a 415-bp *hpt-*specific probe, PCR-labeled with digoxigenin (DIG). Hybridization and probe detection were performed using a DIG Luminescent Detection Kit for Nucleic Acids (Roche Diagnostics, UK) per the manufacturer’s protocol.

Correlation analysis was conducted to investigate relationships between disease resistance percentages and relative gene expression (fold change) across 11 *MusaVicilin* transgenic lines and NTC plants. The relative gene expression levels were quantified using qRT-PCR and normalized relative to the NTC (Livak and Schmittgen, 2001). Disease resistance was assessed as a percentage as previously described by Tripathi et al. (2014a). Pearson’s correlation analysis was conducted to determine the linear relationships between the two variables using the cor() function in R software (version 4.4.3). The statistical significance of the pairwise correlations was evaluated using the cor.test() function, and a matrix of p-values was generated using the cor_pmat() function. The results were visualized in a matrix format using ggcorrplot. Prior to analysis, data distributions were examined for normality to justify the use of Pearson correlation. Statistical significance was evaluated at p ≤ 0.05. Each transgenic line and NTC were treated as independent data points (n=12). Correlations were interpreted to identify potential associations between transgene expression, and resistance phenotype.

### 2.11 Statistical analysis

Minitab Statistical Software, version 17 (Pennsylvania, USA), was used to analyze the data. One-way analysis of variance (ANOVA) was used to compare the differences in disease resistance and plant growth traits across different transgenic lines and NTC plants. Fisher’s HSD test was used to determine the significant difference between the means, and statistical significance was determined at p ≤ 0.05. Correlation analyses between gene expression levels and phenotypic traits were performed using R software (version 4.4.3).

## 3 Results

### 3.1 Bioinformatics analysis and identification of MusaVicilin conserved domains

**Figure 1:**
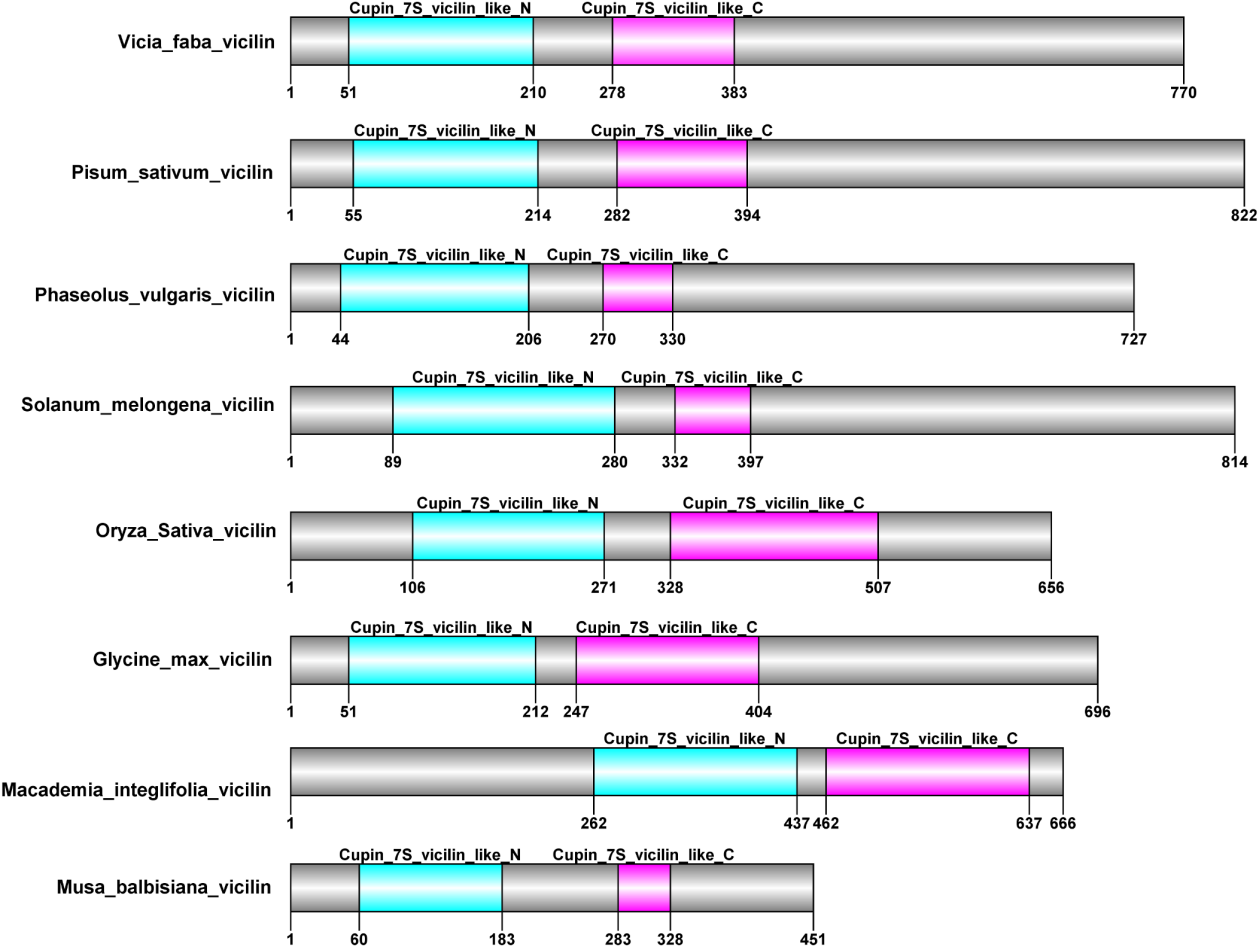
Identification of conserved Vicilin domain in MusaVicilin relative to well characterized legume Vicilin proteins. Blue shade - cupin 7S vicilin N-terminal domain, red shade - cupin 7S vicilin C-terminal domain, Grey – no domain protein regions. Numbers below each protein correspond to the actual length of the protein.

Comparative analysis of the conserved domain of MusaVicilin relative to the vicilin proteins from six legumes and the monocot rice in the CDD database revealed a high degree of structural domain conservation, particularly the canonical vicilin 7S cupin domains (**Figure 1**, **Supplementary Table 1**). Conserved amino acid residues associated with vicilin structural and functional conservation were observed to be present within the Cupin_7S_N and C domains in MusaVicilin as well as all the other vicilins.

The domain conservation was also similarly observed in rice, an equally distant species to the legumes further affirming that MusaVicilin is rightfully a 7S vicilin protein. Despite the conserved 7s domains observed in all the vicilins, two major differences were also observed: 1) variation in overall protein length and 2) differences in individual domain lengths. The overall protein length ranged from 451 aa in MusaVicilin to 822 aa in *Pisum sativum* vicilin.

### 3.2 Phylogenetic analysis of MusaVicilin

The BLASTp query of legume vicilins using MusaVicilin resulted in a total of 104 protein sequences that were successfully downloaded for use in phylogenetic analysis. Out of the 104 sequences, 12 sequences (**Supplementary Table 2**) did not meet the trimming criteria within the alignment pipeline and were therefore dropped in the final list.

**Figure 2:**
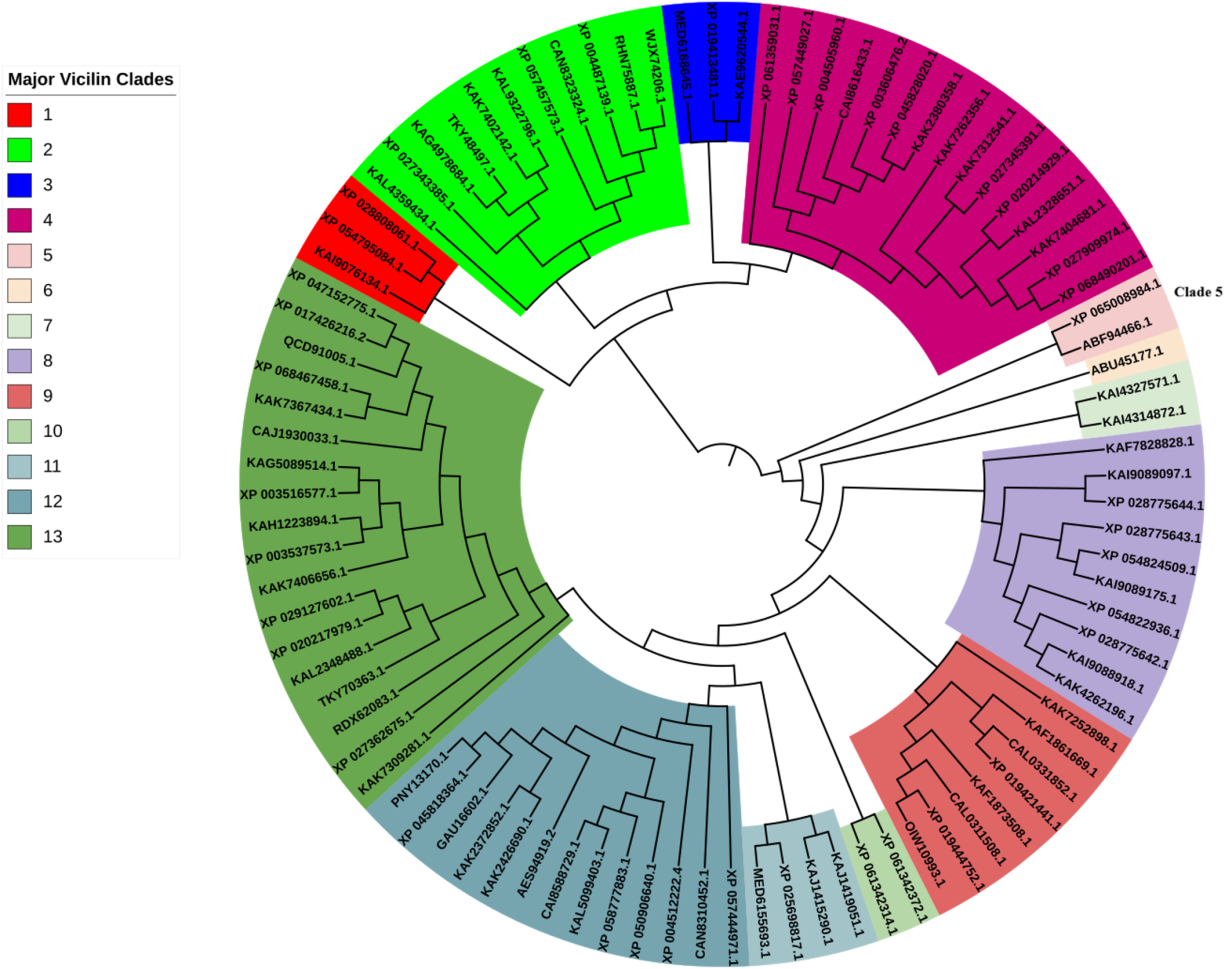
Phylogenetic analysis of MbVicilin protein with dicot Vicilins. Different colours represent clades and subclades with bootstrap support values of >95%.

The maximum-likelihood phylogenetic tree resulted in a total of 13 well bootrap supported clades and subclades. The MusaVicilin protein (XP_065008984.1) was expectedly in sub-clade number 5 with rice vicilin (ABF94466.1) (**Figure 2**). The sub-clade number 5 was closely associated with single protein clade containing the highly characterized vicilin from eggplant (ABU45177.1). This result implies that despite the evolutionary divergence between banana (a monocot) and legumes, MusaVicilin still retains key features of the vicilin protein family. These findings indicate that MusaVicilin likely shares functional and structural characteristics with canonical dicot vicilins, supporting its annotation as a bona fide vicilin family member.

### 3.3 *MusaVicilin* plasmid construct

**Figure 3:**
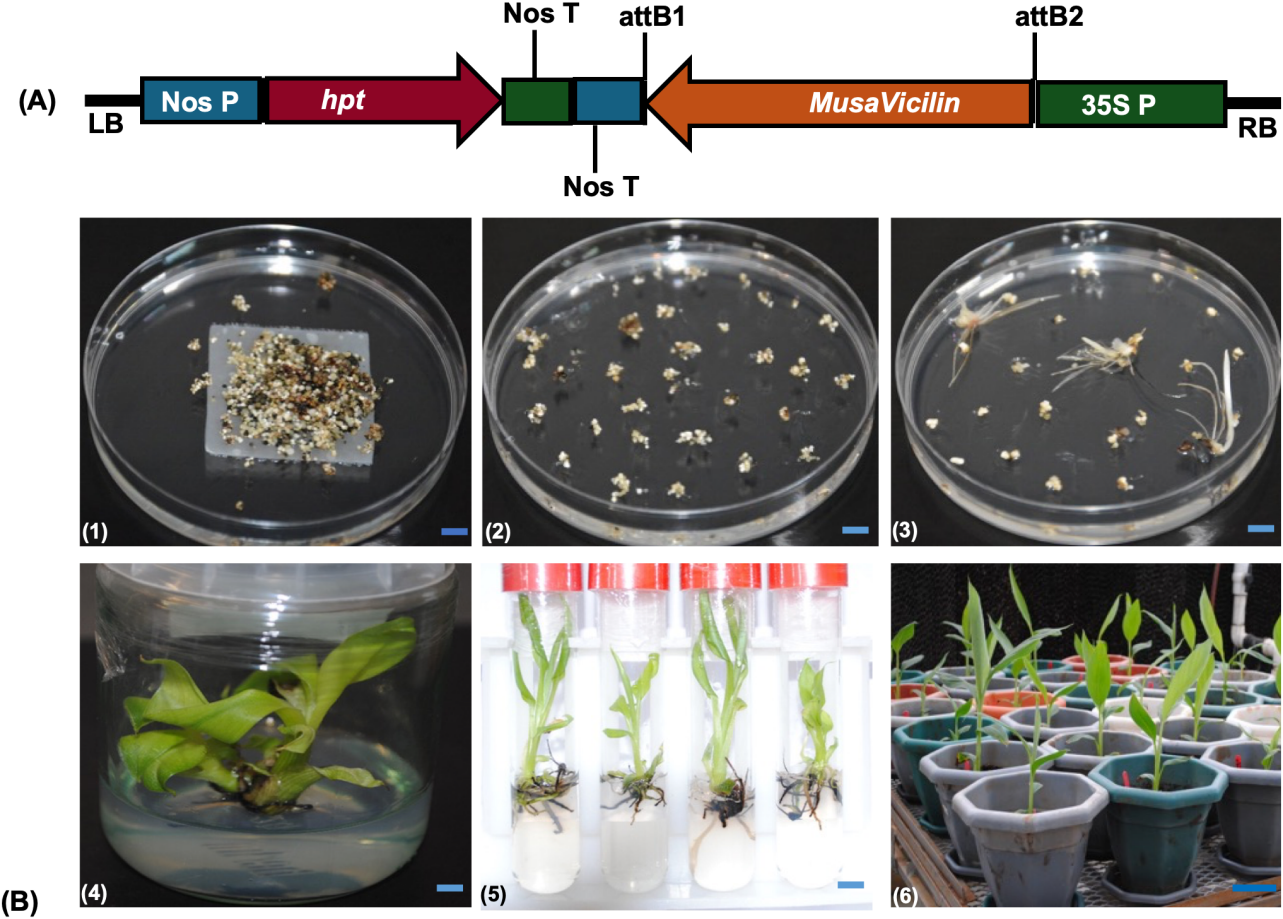
Schematic presentation of construct (pMDC32_MbVicilin) and transgenic lines generation. **(A)** pMDC32_MbVicilin plasmid construct, LB; left border, Nos P; Nopaline Synthase Promoter, hpt; hygromycin phosphotransferase selectable marker gene, Nos T; Nopaline Synthase Terminator, 35S P; Cauliflower Mosaic Virus (CaMV) 35S promoter, RB right border. **(B)** Generation of transgenic lines, (1) Agro-infected embryogenic cells on selective embryo development medium; (2) Embryos maturing on selective embryo maturation medium; (3) Germination of embryos in selective embryo germination medium; (4) Multiplication of plantlets in proliferation medium; (5) Well-rooted plantlets in proliferation medium; (6) Potted transgenic lines in the greenhouse awaiting disease assay. The scale bar represents 1 cm for panels (1) – (5), and 10 cm for panel (6).

The *MusaVicilin* gene (ITC1587_Bchr3_P08153; Mba01_g02390.1) was successfully amplified from *M. balbisiana* using PCR primers (Mb_Vicilin_F/R), yielding a 1,664 bp amplicon. Cloning into the Gateway entry vector pCR8/GW/TOPO and subsequent LR recombination into the binary vector pMDC32 generated the plasmid construct pMDC32*_MbVicilin* (**Figure 3A**), verified by colony PCR, *EcoR*I digestion, and Sanger sequencing. The construct, harboring the *MusaVicilin* gene under the *CaMV* 35S promoter and hygromycin selection marker, was confirmed in *Agrobacterium tumefaciens* EHA105 via colony PCR.

### 3.4 Generation of transgenic lines

‘Sukali Ndiizi’ ECSs were successfully transformed with *Agrobacterium tumefaciens* strain EHA105 containing the pMDC32-35S::*MbVicilin* plasmid. The transformed embryogenic cells were positively selected on hygromycin 12.5 mg/L-containing medium, whereas untransformed cells turned black (**Figure 3B** (1-2)). A total of 52 independent lines were generated. Transgenic lines were multiplied (**Figure 3C**-E3B(4-5), then successfully developed roots (**Figure 3B**(5)) within 3–4 weeks across all independent lines. Following acclimatization, well-rooted plantlets of the transgenic lines were established in the greenhouse for growth analysis and disease evaluation (**Figure 3B**(6)). Additional transgenic lines were maintained in proliferation medium (PM) under 16 h/8 h photoperiod at 26–28 °C, subculturing every 6–8 weeks.

### 3.5 PCR analysis of transgenic lines

**Figure 4:**
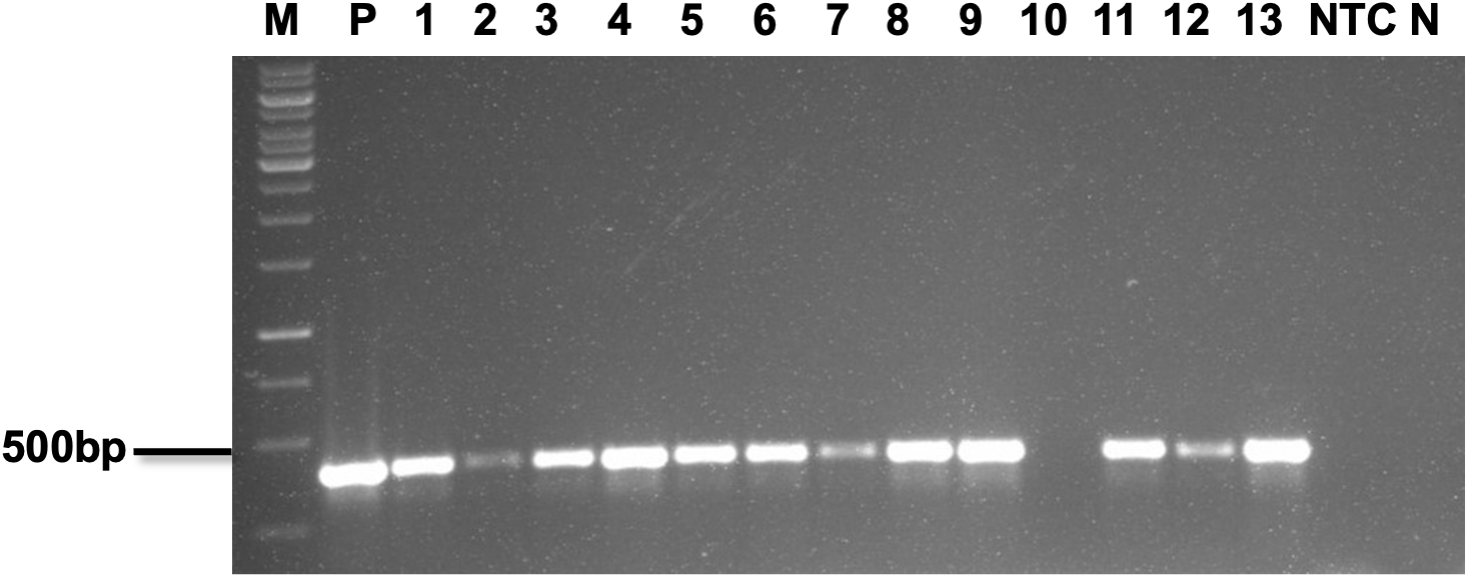
Molecular analysis of putative transgenic banana lines. Representative polymerase chain reaction assay to confirm the presence of hpt gene, M;1kb DNA ladder, 1-13; transgenic lines, P; Plasmid, NTC; Non-transgenic control, N; non-template control.

The 52 regenerated putative *MusaVicilin* transgenic lines were validated for the presence of the *hpt* transgene using PCR with *hpt*-specific primers. All the tested lines showed the expected 415 bp amplicon, confirming successful transgenesis (**Figure 4**). No amplification was observed in the NTC plants.

### 3.6 Plant growth analysis of transgenic lines

**Figure 5:**
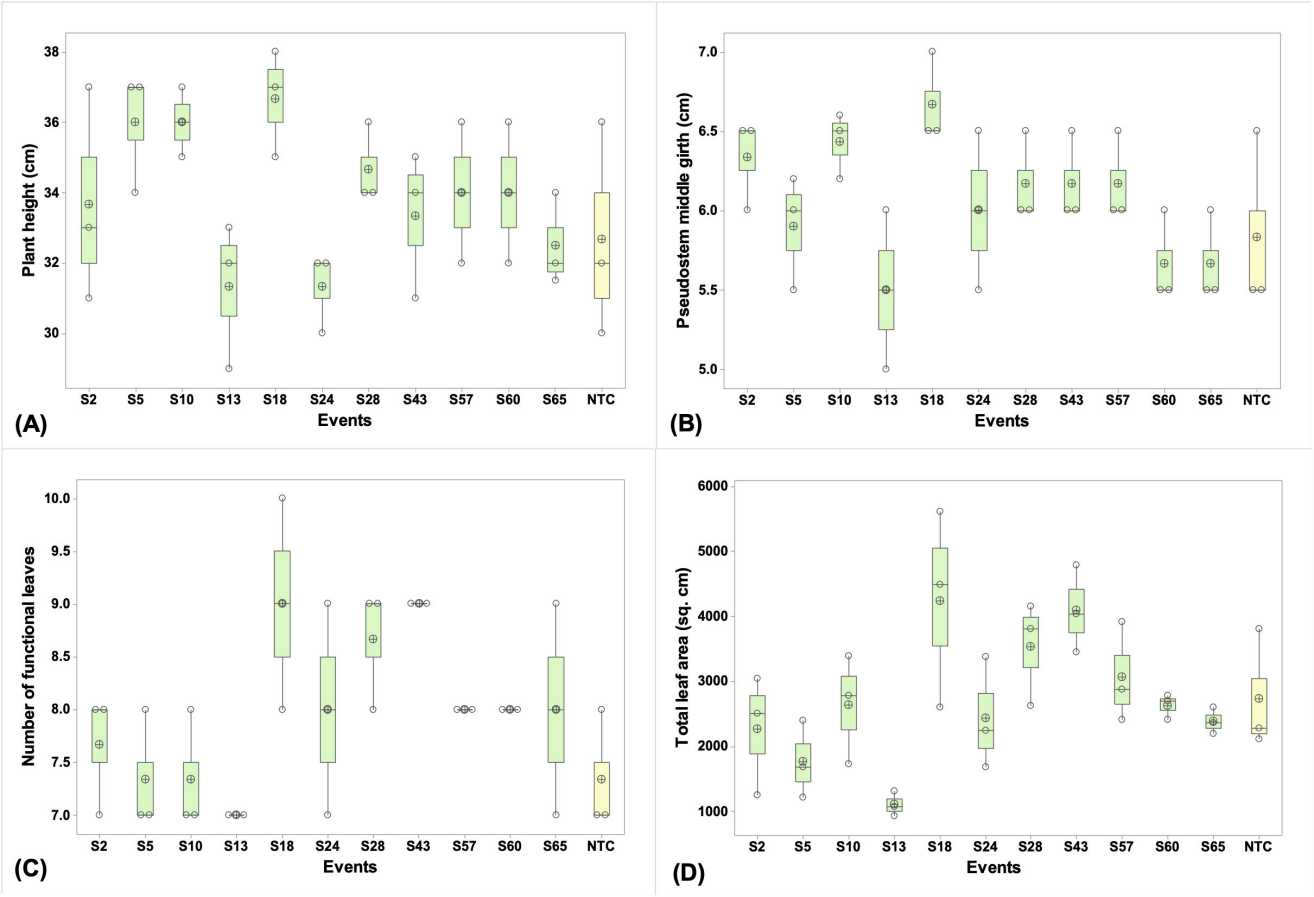
Growth analysis of banana transgenic lines. **(A)** Plant height, **(B)** Pseudostem girth at middle, **(C)** Number of functional leaves, **(D)** Total leaf area. Data were presented as means ± standard deviation. Bars with different letters are significantly different at p ≤ 0.05 according to Fisher’s HSD test (n = 3).

To evaluate the impact of overexpression of *MusaVicilin* gene on plant morphology, growth parameters were assessed across all 52 PCR-positive transgenic lines. Representative data from 11 lines spanning diverse growth phenotypes (S2, S5, S10, S13, S18, S24, S28, S43, S57, S60, S65), are shown in **Figure 5**, demonstrating comparable growth to NTC plants. No morphological defects were detected, and all the *MusaVicilin* lines grew normally. Also, no significant differences (p ≤ 0.05) were observed in the different growth parameters between the transgenic lines and the NTC plants, suggesting that overexpression of the *MusaVicilin* gene in the transgenic lines did not cause any adverse growth effect.

### 3.7 Evaluation of transgenic lines for disease resistance

**Figure 6:**
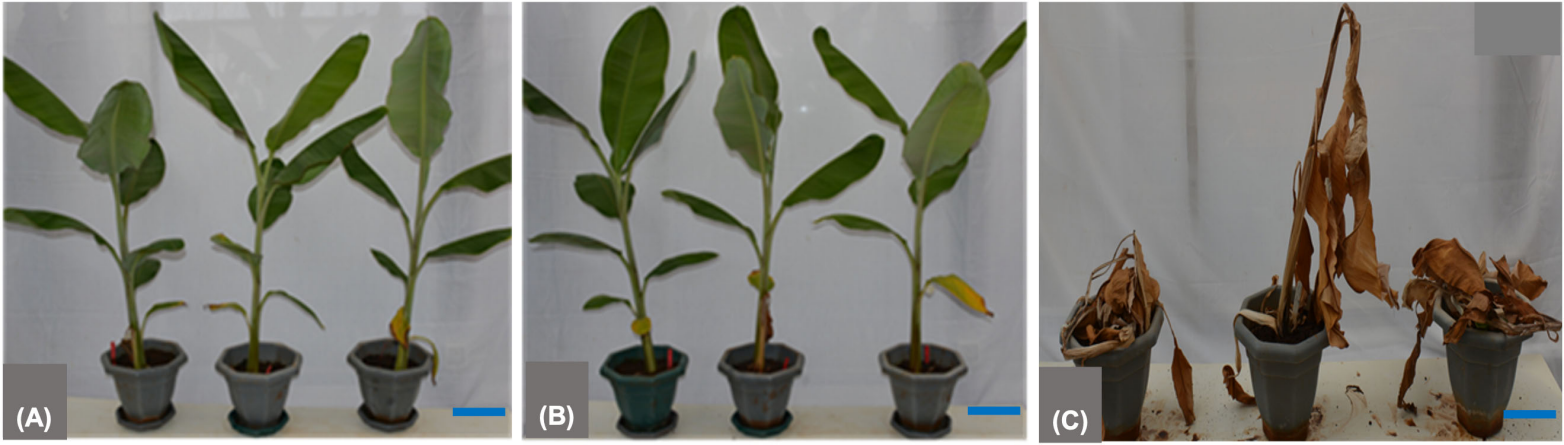
Disease evaluation of banana transgenic lines against Xanthomonas vasicola pv. musacearum. **(A)** Transgenic banana line S2 showing complete resistance to BXW, **(B)** Line S57 showing wilting symptom solely on the inoculated leaf of the second replicate plant, **(C)** Non-transgenic control exhibiting complete wilting. Plants were assessed for 60 days post inoculation with Xvm. The scale bar represents 10 cm.

To assess the response of transgenic lines to the bacterial pathogen *Xvm*, 52 lines, along with NTC plants, underwent evaluation for disease resistance under greenhouse conditions. The NTC plants exhibited pronounced symptoms (chlorosis or necrosis) after about 16 dpi, which spread throughout the entire plant, culminating in complete wilting at 22.67 ± 4.93 dpi (**Table 1**) with a 100% disease incidence (**Figure 6C**). This confirmed their susceptibility to *Xvm* infection. In contrast, line S2 exhibited complete resistance against *Xvm* (**Figure 6A**), displaying no disease symptoms upon *Xvm* challenge (**Table 1**). Additionally, line S5 (**Figure 6B**) demonstrated a substantial disease resistance (95.2%), with the manifestation of disease symptoms limited solely to the inoculated leaf with a very low disease severity index of 4.8%. The remaining 50 lines showed partial resistance against *Xvm*, ranging from 25% to 77.8%, with only about 15% of the lines showing susceptibility comparable to that of the NTC plants. Disease severity scores ranged from 0 ± 0 (S2) to 5 ± 0 (S43, S65, and NTC). Fisher LSD grouping (p<0.05) distinguished resistant line S2 (0 ± 0), along with S5, S24, and S57 (0–1.33), from susceptible NTC (5 ± 0), confirming multiple transgenic lines with significantly reduced disease severity. DSI values correspondingly spanned 0–33.3% across the *MusaVicilin* lines versus 100% in NTC, where lines S43, S65 clustered with controls at maximum severity (**Table 1**). This comprehensively unveils a spectrum of disease resistance levels among the *MusaVicilin* lines, signifying the efficacy of constitutive overexpression of the *MusaVicilin* gene in conferring protection against *Xvm* infection.

**Table 1:** Greenhouse evaluation of transgenic lines for resistance against *Xanthomonas vasicola* pv *musacearum*.

Line Number	Mean number of days to the appearance of first symptom	Mean number of days to complete wilting	Disease severity (mean ± std)	Disease Severity Index (%)	Average resistance (%)	Disease rating
S2	NS	NW	0 ± 0^c^	0	100 ± 0^a^	R
S5	58 ± 3.46^a^	NW	0.33 ± 0.47^bc^	6.7	95.24 ± 8.25^a^	PR
S10	49.7 ± 17.9^a^	55.67 ± 7.51^a^	1.67 ± 2.36^ab^	33.3	66.7 ± 57.7^ab^	PR
S13	46.3 ± 23.7^ab^	49.33 ± 18.5^ab^	1.67 ± 2.36^ab^	33.3	66.7 ± 57.7^ab^	PR
S18	51.67 ± 14.43^a^	57.67 ± 4.04^a^	1.67 ± 2.36^ab^	33.3	66.7 ± 57.7^ab^	PR
S24	48 ± 20.8^a^	NCW	1.33 ± 1.89^bc^	26.7	72.2 ± 48.1^a^	PR
S28	44 ± 27.7^ab^	49.33 ± 18.5^ab^	1.67 ± 2.36^ab^	33.3	66.7 ± 57.7^ab^	PR
S43	21 ± 3.61^bc^	46 ± 6.08^ab^	5 ± 0.0^a^	100	0 ± 0^b^	S
S57	52.67 ± 12.70^a^	NCW	1 ± 1.41^bc^	20	77.8 ± 38.5^a^	PR
S60	48 ± 20.8^a^	53 ± 12.12^a^	1.67 ± 2.36^ab^	33.3	66.7 ± 57.7^ab^	PR
S65	17.67 ± 2.31^c^	34.33 ± 1.155^bc^	5 ± 0.0^a^	100	0 ± 0^b^	S
NTC	16.33 ± 3.79^c^	22.67 ± 4.93^c^	5 ± 0.0^a^	100	0 ± 0^b^	S

Means followed by the same superscript letter in the same column are not significantly different according to Fisher HSD test at p ≤ 0.05.

Data were presented as means ± standard deviation (SD).

NS, no symptom; NW, no wilting; R, Resistant; PR, Partial Resistance; S, Susceptible.

### 3.8 Relative expression of the *MusaVicilin* gene

**Figure 7:**
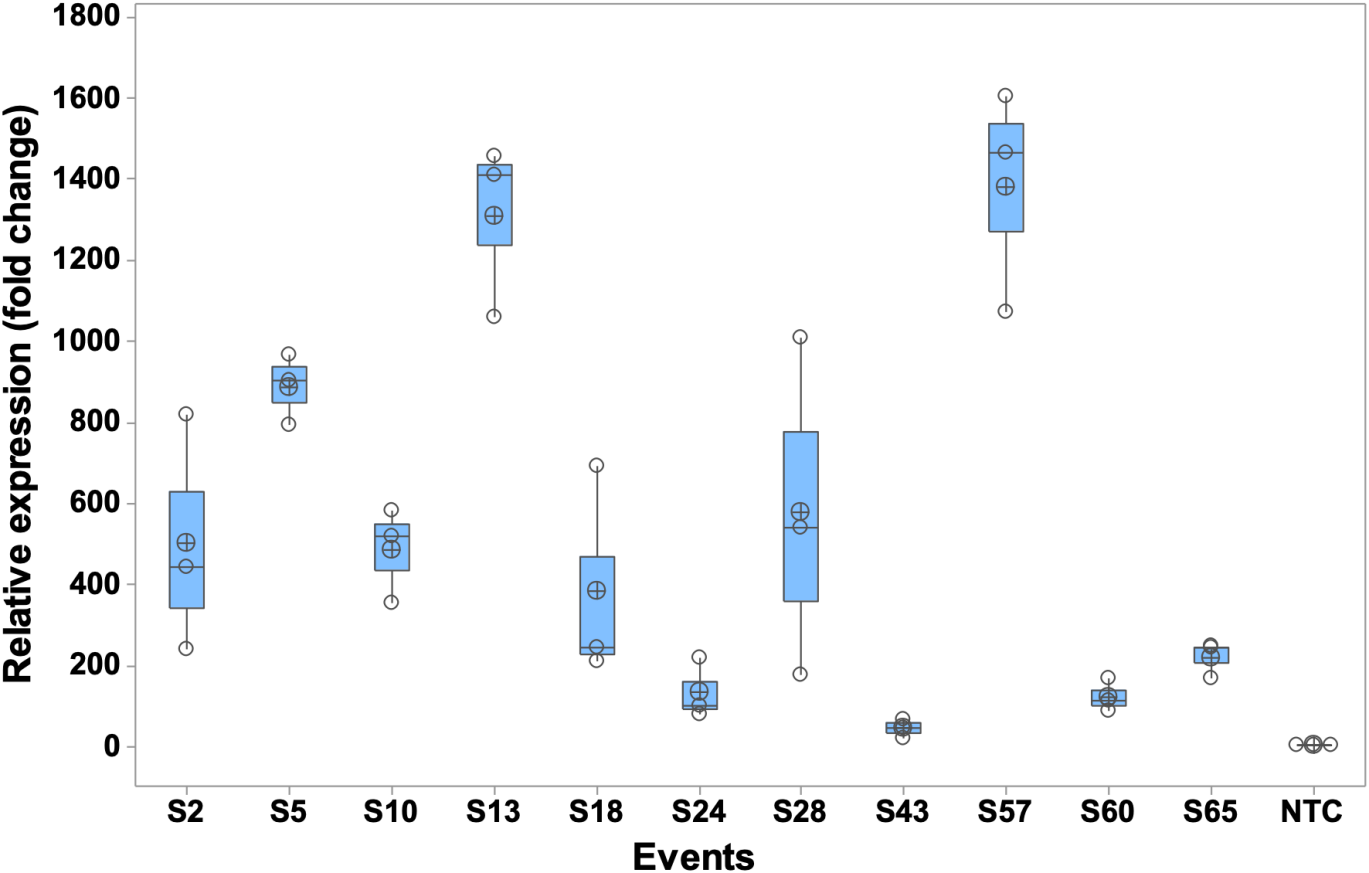
Relative expression of *MusaVicilin* gene in transgenic lines. qRT-PCR analysis of constitutively overexpressed *MusaVicilin* gene in eleven transgenic lines and non-transgenic control (NTC). The boxplots represent mean±SE (n-3 biological replicates) normalized to the *Musa 25S* gene.

Eleven transgenic lines (S2, S5, S10, S13, S18, S24, S28, S43, S57, S60, S65), representing a range of BXW resistance phenotypes observed in greenhouse screening, were selected for qRT-PCR analysis of the *MusaVicilin* gene. The selected lines represented a range of tolerance levels from 0% (non-transgenic control) to 100% (**Table 1**). Differences in *MusaVicilin* transcript levels were observed between the transgenic lines investigated compared to NTC. For instance, line S57 exhibited the highest *MusaVicilin* gene expression level (over 1350-fold), followed by lines S13 and S5. Line S43 exhibited the least fold of relative expression (120-fold) (**Figure 7**). These results confirm successful overexpression of the *MusaVicilin* gene in the transgenic lines.

### 3.9 Southern blot analysis

**Figure 8:**
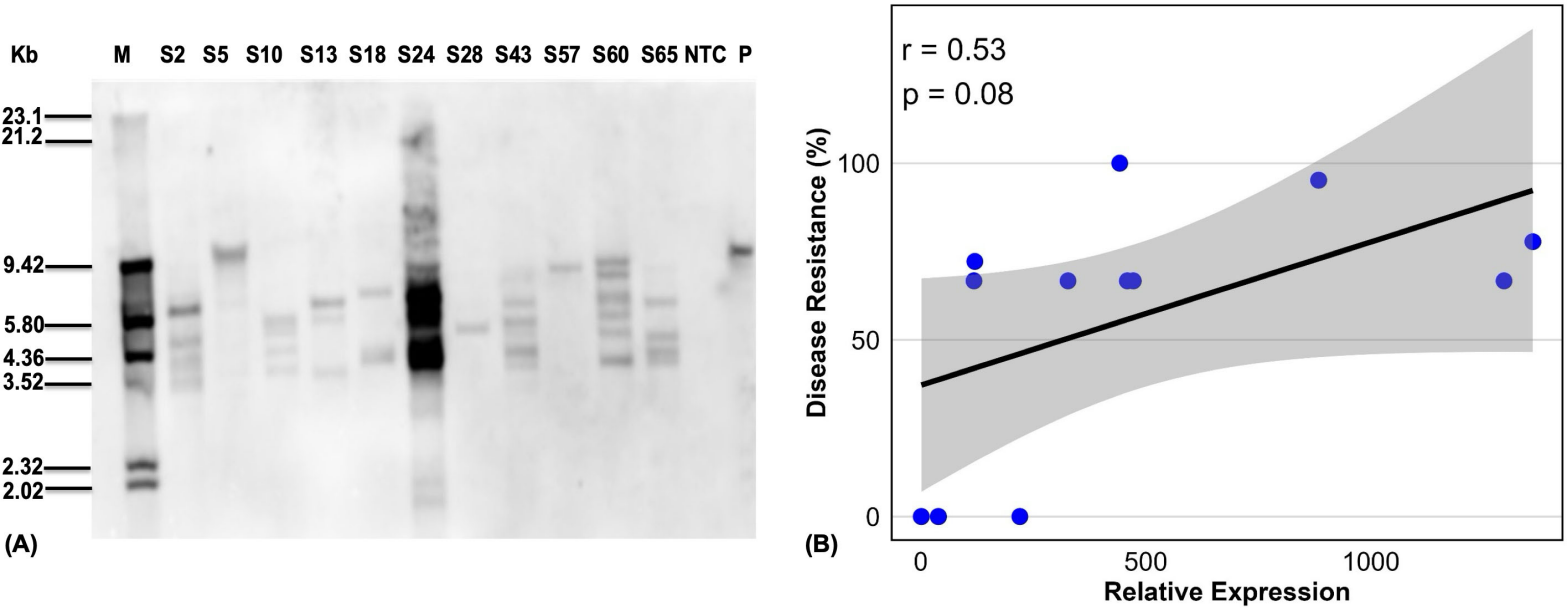
Southern blot and Pairwise Pearson correlation analysis. **(A)** Southern blot analysis to confirm the integration of *transg*ene showing copy number, M; molecular marker, NTC; Non-transgenic control, P; plasmid. **(B)** Pairwise Pearson correlation analysis of transgenic lines. Scatter plot illustrating the Pearson correlation between disease resistance percentages and relative gene expression (fold change) in *MusaVicilin* lines. A moderate positive correlation was observed (r = 0.53), although it was not statistically significant (p = 0.08). Data points are distributed along an upward-sloping diagonal line, indicating that increased gene upregulation via constitutive overexpression is associated with enhanced resistance levels.

To verify transgene integration and copy number, Southern blot analysis was performed on 11 transgenic lines (S2, S5, S10, S13, S18, S24, S28, S43, S57, S60, S65) representing diverse BXW resistance profiles from greenhouse screening assays. Southern blot hybridization of *Hind*III-digested genomic DNA using *hpt* probe confirmed the integration of the transgene in the plant genome with different hybridization profiles, indicating random insertion of the transgene in the genome of the tested lines. The transgene copy number incorporated in the different lines ranged from one to multiple (**Figure 8A**). Lines S5, S28, and S57 had a single gene copy, with lines S13 and S18 having three gene copies, while line S60 had the most gene copies. No transgene integration was detected in the NTC plant.

Correlation analysis between *MusaVicilin* expression levels and BXW resistance across the 11 transgenic lines revealed a moderate positive trend (Pearson’s r = 0.53, p = 0.08; **Figure 8B**). This suggests that lines with higher *MusaVicilin* expression tended to exhibit greater resistance to *Xvm* infection. Although events with higher expression tended toward greater resistance, the relationship was not statistically significant (p > 0.05), suggesting additional factors influence resistance outcomes.

## 4 Discussion

BXW poses a significant threat to banana production in East and Central Africa, impacting millions of livelihoods and regional food security. Conventional management strategies, including cultural practices and breeding for disease resistance have had limited success in controlling BXW due to the genetic complexity of banana breeding and the limited availability of resistant traits (Batte et al., 2019). The absence of fertile diploid bananas completely resistant to BXW further limits traditional breeding efforts (Tripathi et al., 2009, 2014a; Namukwaya et al., 2012). To overcome these challenges, modern biotechnological approaches such as genetic transformation offer promising alternatives by introducing and overexpressing resistance genes to develop BXW-resistant banana varieties.

Bioinformatics analysis identified MusaVicilin (MbVicilin; XP_065008984.1) as a genuine vicilin protein. Sequence comparisons showed conserved Vicilin domains and key residues characteristic of cupin 7S superfamily (cl40423), a defining feature of plant 7S storage proteins (Dunwell et al., 2000). Phylogenetic reconstruction further grouped MusaVicilin with well-characterized vicilin proteins further affirming family membership across monocot-dicot divergence (Shewry, 1995). Vicilin storage proteins are known precursors of cysteine-rich antimicrobial peptides released during seed germination (Xie et al., 2016), suggesting potential AMP functionality for MusaVicilin. This provides a rationale for its selection for developing resistance to BXW disease.

Antimicrobial peptides (AMPs) are crucial plant defense molecules with broad activity against pathogens (Broekaert et al., 1997; Ryan et al., 2007; Tam et al., 2015). Vicilin-like proteins release potent AMPs that provide effective protection against various disease-causing pathogens and pests (Gomes et al., 1998; Marcus et al., 2008; Gil-Salido et al., 2024). The upregulation of the *MusaVicilin* gene, a vicilin-like AMP, during BXW infection in resistance to *Musa balbisiana* at early infection, further supports its role in plant defense (Tripathi et al., 2019). By overexpressing the *MusaVicilin* gene in the BXW-susceptible banana, the colonization of *Xvm* could be restricted. We confirmed this hypothesis by constitutively overexpressing the *MusaVicilin* gene in the susceptible banana cultivar ‘Sukali Ndiizi’, and the transgenic lines generated were evaluated for their response against *Xvm* under greenhouse conditions. These assays revealed that transgenic banana overexpressing *MusaVicilin* exhibited enhanced resistance to BXW disease compared to NTC plants.

The *MusaVicilin* transgenic lines demonstrated a spectrum of resistance against *Xvm* infection, underscoring its potential in bacterial wilt defense. The complete resistance observed in line S2 (0 ± 0 severity), coupled with the near-complete resistance in line S5 (0.33 ± 0.47 severity, 95.2%, 6.7% DSI), highlights the capacity of the *MusaVicilin* gene to block pathogen progression when stably expressed at optimal levels effectively. This aligns with studies in *Capsicum baccatum L.* (Vieira Bard et al., 2014)*, Clitoria fairchildiana* (Bertonceli et al., 2022), *Macadamia integrifolia*, and *Theobroma cacao* (Marcus et al., 1999)*, Centrosema virginianum* (Xie et al., 2016)*, Enterolobium contortisiliquum* (Moura et al., 2007), and cowpea (Sales et al., 2001; Almeida et al., 2022), where vicilin-like AMPs disrupted bacterial cell membranes or suppressed virulence factors, thereby curtailing disease spread. Partial resistance (25% - 75%) observed in the remaining lines (S24: 1.33 ± 1.89 severity, 26.7% DSI; S57: 1 ± 1.41 severity, 20% DSI) suggested that transgene expression levels, copy number, or insertion sites critically influence gene efficacy, consistent with findings in other AMP-overexpression systems (Tam et al., 2015). The non-transgenic control plants wilted rapidly (22.67 ± 4.93dpi, 5 ± 0 severity, 100% DSI), compared to the resistant lines that delayed pathogen colonization, comparable with other vicilin-like AMPs (Lay and Anderson, 2005). Although 15% of lines remained susceptible, most showed improved disease resistance compared to NTC plants, confirming the potential of the *MusaVicilin* gene in bacterial wilt management. The observed variation in resistance levels among the transgenic lines likely reflects positional effects of transgene insertion, which can significantly influence gene expression and stability (Gelvin, 2017). Integration near heterochromatic regions, transcriptionally inactive zones, or regulatory elements may lead to variable or silenced expression, thereby impacting the resistance phenotype. Hence, thorough molecular characterization and screening of multiple independent transformation lines are essential in understanding and managing positional effects, ensuring the successful development of stable and effective transgenic disease-resistant plants (Guttikonda et al., 2016; Giraldo et al., 2020).

The *MusaVicilin* transcript levels in the transgenic lines varied widely and did not consistently correlate with transgene copy number. For instance, line S2, with four gene copies, exhibited approximately 400-fold overexpression, achieving complete resistance to *Xvm*, whereas line S60, despite having six gene copies, showed only a 220-fold overexpression and no resistance. Similarly, lines S57 (77.8%) and S13 (66.7%) demonstrated extremely high expression levels (up to 1,300-fold) with just one and three gene copies, respectively, while line S24, carrying five gene copies, had a comparatively low 120-fold expression. Line S43, with four copies, showed a mere 4-fold increase in transcript levels and succumbed to the disease. This confirms the moderate positive correlation between gene expression and disease resistance observed (r = 0.53, p = 0.08), suggesting that increased transgene expression may enhance resistance, although this relationship was not statistically significant. There was no linear relationship between transgene copy number and expression levels or disease resistance. Such discrepancies suggest that transgene copy number alone does not determine expression or resistance. Instead, factors such as positional effects of transgene insertion (Gelvin, 2017), promoter activity (Villao-Uzho et al., 2023), transcriptional silencing (Matzke et al., 1994), and epigenetic regulation (Matzke and Matzke, 1998) likely play critical roles in modulating expression levels. Moreover, the threshold of *MusaVicilin* expression required for effective defense against BXW may not be linearly related to copy number but rather to the efficiency of transcript accumulation and subsequent protein activity. These findings highlight key points, especially that successful resistance requires achieving sufficiently high *MusaVicilin* expression, regardless of copy number, and future work should focus on optimizing expression stability, possibly through targeted transgene integration, promoter engineering, or genome-editing approaches.

Overexpression of the *MusaVicilin* gene in the ‘Sukali Ndiizi’ banana cultivar, did not induce noticeable adverse effects on plant morphology under greenhouse conditions. Transgenic lines were phenotypically indistinguishable from NTC plants, suggesting that *MusaVicilin* overexpression may not negatively impact yield, aligning with findings in other crops like cucumber, cotton, brassica, and soybean (Bakare et al., 2022; Tang et al., 2023; Derbyshire et al., 2024; Wang et al., 2024). However, these results were obtained under controlled greenhouse conditions, and confined field trials are necessary for a definitive assessment of the gene’s impact on agronomic traits and overall productivity under realistic growing conditions.

Antimicrobial peptides (AMPs) slow disease progression by disrupting the membranes of pathogenic cells, inhibiting enzyme activities, and interfering with pathogen replication (Benfield and Henriques, 2020). These mechanisms reduce pathogen load and slow down the spread of infection within the plant. AMPs contribute to both basal and non-host disease resistances by providing a broad-spectrum defense against a wide range of pathogens (Nayab et al., 2022). This dual role is reflected in their ability to enhance quantitative resistance by delaying symptom appearance and reducing disease severity, as well as providing qualitative resistance by entirely preventing pathogen infection in some cases (Cook, 1998; Dodds and Rathjen, 2010; Gill et al., 2015). In this study, the overexpression of the *MusaVicilin* gene in the transgenic lines demonstrated quantitative resistance to BXW. Specifically, these transgenic lines showed delayed disease symptom onset ranging up to 58 days, compared to 12 days for NTC plants. Furthermore, the transgenic lines exhibited a lower disease severity (38.3%), compared to 100% for NTC plants. These findings demonstrate that *MusaVicilin* overexpression enhances quantitative resistance to BXW, providing a promising approach for banana disease management.

While 4 out of 52 transgenic lines exhibited ≥70% resistance to BXW, several limitations warrant consideration. The modest correlation between *MusaVicilin* expression and resistance (r=0.32, p=0.15) suggests the presence of threshold effects, where resistance may plateau once protein levels exceed a critical level. Greenhouse screening provides initial evidence of resistance but needs to be further tested in confined field trials under natural pathogen pressure and diverse agro-ecological conditions. Finally, further studies are required to evaluate the stability and heritability of resistance in subsequent generations.

In summary, this study demonstrates that transgenic banana overexpressing the *MusaVicilin* antimicrobial peptide gene exhibit enhanced resistance against *Xvm*, providing a strong foundation for engineering durable resistance to BXW. The synergistic stacking of *MusaVicilin* with other defense-related genes could further strengthen and broaden resistance, offering a more robust defense strategy. Further evaluation under confined field trials is essential to validate their disease resistance efficacy and agronomic performance.

## Data Availability

The original contributions presented in the study are included in the article/**Supplementary Material**. Further inquiries can be directed to the corresponding author.

## References

[B1] Abele S. Pillay M. (2007). Bacterial wilt and drought stresses in banana production and their impact on economic welfare in Uganda: Implications for banana research in east African highlands. J. Crop Improv. 19, 173–191. doi: 10.1300/j411v19n01_09, PMID: 17855243

[B2] Abele S. Twine E. Legg C. (2007). Food security in Eastern Africa and the Great Lakes. Available online at: https://biblio.iita.org/documents/U07RepAbeleFoodNothomNodev.pdf-7030b208048ef052c0d624784a004a83.pdf (Accessed October 14, 2025).

[B3] Adero M. Tripathi J. N. Oduor R. Zipfel C. Tripathi L. (2023). Transgenic expression of Arabidopsis ELONGATION FACTOR-TU RECEPTOR (AtEFR) gene in banana enhances resistance against Xanthomonas campestris pv. musacearum. PloS One 18, e0290884. doi: 10.1371/journal.pone.0290884, PMID: 37656732 PMC10473477

[B4] Almeida T. S. da Cruz Souza C. A. de Cerqueira e Silva M. B. Batista F. P. R. Ferreira E. S. Santos A. L. S. . (2022). Extraction and characterization of β-viginin protein hydrolysates from Cowpea flour as a new manufacturing active ingredient. Technol. (Basel) 10, 89. doi: 10.3390/technologies10040089, PMID: 41725453

[B5] Bailey C. J. Boulter D. (1972). The structure of vicilin of Vicia faba. Phytochemistry 11, 59–64. doi: 10.1016/S0031-9422(00)89967-7, PMID: 41334505

[B6] Bakare O. O. Gokul A. Fadaka A. O. Wu R. Niekerk L.-A. Barker A. M. . (2022). Plant antimicrobial peptides (PAMPs): Features, applications, production, expression, and challenges. Molecules 27, 3703. doi: 10.3390/molecules27123703, PMID: 35744828 PMC9229691

[B7] Batte M. Swennen R. Uwimana B. Akech V. Brown A. Tumuhimbise R. . (2019). Crossbreeding East African highland bananas: Lessons learnt relevant to the botany of the crop after 21 years of genetic enhancement. Front. Plant Sci. 10. doi: 10.3389/fpls.2019.00081, PMID: 30804965 PMC6370977

[B8] Benfield A. H. Henriques S. T. (2020). Mode-of-action of antimicrobial peptides: Membrane disruption vs. Intracellular mechanisms. Front. Med. Technol. 2. doi: 10.3389/fmedt.2020.610997, PMID: 35047892 PMC8757789

[B9] Bertonceli M. A. A. Oliveira A. E. A. Ferreira A. T. S. Perales J. Fernandes K. V. S. (2022). A vicilin-like protein extracted from Clitoria fairchildiana cotyledons was toxic to Callosobruchus maculatus (Coleoptera: Chrysomelidae). Pestic. Biochem. Physiol. 184, 105129. doi: 10.1016/j.pestbp.2022.105129, PMID: 35715067

[B10] Biruma M. Pillay M. Tripathi L. Blomme G. Abele S. Mwangi M. . (2007). Banana Xanthomonas wilt: a review of the disease, management strategies and future research directions. Afr. J. Biotechnol. 6, 953–962. doi: 10.4314/AJB.V6I8.56989

[B11] Bollini R. Chrispeels M. J. (1978). Characterization and subcellular localization of vicilin and phytohemagglutinin, the two major reserve proteins of Phaseolus vulgaris L. Planta 142, 291–298. doi: 10.1007/BF00385080, PMID: 24408192

[B12] Broekaert W. F. Cammue B. P. A. De Bolle M. F. C. Thevissen K. De Samblanx G. W. Osborn R. W. . (1997). Antimicrobial peptides from plants. CRC Crit. Rev. Plant Sci. 16, 297–323. doi: 10.1080/07352689709701952, PMID: 41799851

[B13] Christelová P. Valárik M. Hřibová E. De Langhe E. Doležel J. (2011). A multi gene sequence-based phylogeny of the Musaceae (banana) family. BMC Evol. Biol. 11, 103. doi: 10.1186/1471-2148-11-103, PMID: 21496296 PMC3102628

[B14] Cook R. J. (1998). The molecular mechanisms responsible for resistance in plant-pathogen interactions of the gene-for-gene type function more broadly than previously imagined. Proc. Natl. Acad. Sci. U. S. A. 95, 9711–9712. doi: 10.1073/pnas.95.17.9711, PMID: 9707538 PMC33879

[B15] D’Hont A. Denoeud F. Aury J.-M. Baurens F.-C. Carreel F. Garsmeur O. . (2012). The banana (Musa acuminata) genome and the evolution of monocotyledonous plants. Nature 488, 213–217. doi: 10.1038/nature11241, PMID: 22801500

[B16] Derbyshire M. C. Newman T. E. Thomas W. J. W. Batley J. Edwards D. (2024). The complex relationship between disease resistance and yield in crops. Plant Biotechnol. J. 22, 2612–2623. doi: 10.1111/pbi.14373, PMID: 38743906 PMC11331782

[B17] Dodds P. N. Rathjen J. P. (2010). Plant immunity: towards an integrated view of plant-pathogen interactions. Nat. Rev. Genet. 11, 539–548. doi: 10.1038/nrg2812, PMID: 20585331

[B18] Doyle JJ Schuler MA Godette WD Zenger V Beachy RN . (1986). The glycosylated seed storage proteins of Glycine max and Phaseolus vulgaris: Structural homologies of genes and proteins. J. Biol. Chem. 261, 9228–9238. doi: 10.1016/S0021-9258(18)67644-6 3013879

[B19] Dunwell J. M. Khuri S. Gane P. J. (2000). Microbial relatives of the seed storage proteins of higher plants: Conservation of structure and diversification of function during evolution of the cupin superfamily. Microbiol. Mol. Biol. Rev. 64, 153–179. doi: 10.1128/MMBR.64.1.153-179.2000, PMID: 10704478 PMC98990

[B20] FAOSTAT (2023). Available online at: https://www.fao.org/faostat/en/data/QCL (Accessed October 13, 2025).

[B21] Gatehouse J. A. Croy R. R. Morton H. Tyler M. Boulter D. (1981). Characterisation and subunit structures of the vicilin storage proteins of pea (Pisum sativum L.). Eur. J. Biochem. 118, 627–633. doi: 10.1111/j.1432-1033.1981.tb05565.x, PMID: 7297569

[B22] Gelvin S. B. (2017). Integration of Agrobacterium T-DNA into the plant genome. Annu. Rev. Genet. 51, 195–217. doi: 10.1146/annurev-genet-120215-035320, PMID: 28853920

[B23] Gill U. S. Lee S. Mysore K. S. (2015). Host versus nonhost resistance: distinct wars with similar arsenals. Phytopathology 105, 580–587. doi: 10.1094/PHYTO-11-14-0298-RVW, PMID: 25626072

[B24] Gil-Salido A. A. Rojas-Cabeza J. F. Sotelo-Mundo R. R. Islas-Osuna M. A. (2024). “ Role of vicilin in plant defense,” in Defense-Related Proteins in Plants (London, UK: Elsevier), 379–395. doi: 10.1016/b978-0-443-13236-0.00015-4, PMID:

[B25] Giraldo P. A. Shinozuka H. Spangenberg G. C. Smith K. F. Cogan N. O. I. (2020). Rapid and detailed characterization of transgene insertion sites in genetically modified plants via nanopore sequencing. Front. Plant Sci. 11. doi: 10.3389/fpls.2020.602313, PMID: 33613582 PMC7889508

[B26] Gomes V. M. Okorokov L. A. Rose T. L. Fernandes K. V. Xavier-Filho J. (1998). Legume vicilins (7S storage globulins) inhibit yeast growth and glucose stimulated acidification of the medium by yeast cells. Biochim. Biophys. Acta 1379, 207–216. doi: 10.1016/s0304-4165(97)00100-1, PMID: 9528656

[B27] Guttikonda S. K. Marri P. Mammadov J. Ye L. Soe K. Richey K. . (2016). Molecular characterization of transgenic events using next generation sequencing approach. PloS One 11, e0149515. doi: 10.1371/journal.pone.0149515, PMID: 26908260 PMC4764375

[B28] Heslop-Harrison J. S. Schwarzacher T. (2007). Domestication, genomics and the future for banana. Ann. Bot. 100, 1073–1084. doi: 10.1093/aob/mcm191, PMID: 17766312 PMC2759213

[B29] Hoang D. T. Chernomor O. von Haeseler A. Minh B. Q. Vinh L. S. (2018). UFBoot2: Improving the ultrafast bootstrap approximation. Mol. Biol. Evol. 35, 518–522. doi: 10.1093/molbev/msx281, PMID: 29077904 PMC5850222

[B30] I'Anson KJ Miles MJ Bacon JR Carr HJ Lambert N Morris VJ . (1988). Structure of the 7S globulin (vicilin) from pea (Pisum sativum). Int. J. Biol. Macromol. 10, 311–317. doi: 10.1016/0141-8130(88)90010-4

[B31] Iskra-Caruana M.-L. Duroy P.-O. Chabannes M. Muller E. (2014). The common evolutionary history of badnaviruses and banana. Infect. Genet. Evol. 21, 83–89. doi: 10.1016/j.meegid.2013.10.013, PMID: 24184704

[B32] Jain A. Kumar A. Salunke D. (2016). Crystal structure of the vicilin from Solanum melongena reveals existence of different anionic ligands in structurally similar pockets. Sci. Rep. 6, 23600. doi: 10.1038/srep23600, PMID: 27004988 PMC4804240

[B33] Kabasser S. Pratap K. Kamath S. Taki A. C. Dang T. Koplin J. . (2022). Identification of vicilin, legumin and antimicrobial peptide 2a as macadamia nut allergens. Food Chem. 370. doi: 10.1016/j.foodchem.2021.131028, PMID: 34525424 PMC7614219

[B34] Kalyaanamoorthy S. Minh B. Q. Wong T. K. F. von Haeseler A. Jermiin L. S. (2017). ModelFinder: Fast model selection for accurate phylogenetic estimates. Nat. Methods 14, 587–589. doi: 10.1038/nmeth.4285, PMID: 28481363 PMC5453245

[B35] Katoh K. Standley D. M. (2013). MAFFT multiple sequence alignment software version 7: Improvements in performance and usability. Mol. Biol. Evol. 30, 772–780. doi: 10.1093/molbev/mst010, PMID: 23329690 PMC3603318

[B36] Kumar N. Krishnamoorthy V. Nalina L. Soorianathasundharam K. (2002). A new factor for estimating total leaf area in banana. InfoMusa 11, 42–43.

[B37] Lay F. T. Anderson M. A. (2005). Defensins--components of the innate immune system in plants. Curr. Protein Pept. Sci. 6, 85–101. doi: 10.2174/1389203053027575, PMID: 15638771

[B38] Letunic I. Bork P. (2021). Interactive Tree Of Life (iTOL) v5: An online tool for phylogenetic tree display and annotation. Nucleic Acids Res. 49, W293–W296. doi: 10.1093/nar/gkab301, PMID: 33885785 PMC8265157

[B39] Livak K. J. Schmittgen T. D. (2001). Analysis of relative gene expression data using real-time quantitative PCR and the 2(-Delta Delta C(T)) Method. Methods 25, 402–408. doi: 10.1006/meth.2001.1262, PMID: 11846609

[B40] Marcus J. P. Goulter K. C. Manners J. M. (2008). Peptide fragments from plant vicilins expressed in Escherichia coli exhibit antimicrobial activity in *vitro*. Plant Mol. Biol. Rep. 26, 75–87. doi: 10.1007/s11105-008-0024-9, PMID: 41841152

[B41] Marcus J. P. Green J. L. Goulter K. C. Manners J. M. (1999). A family of antimicrobial peptides is produced by processing of a 7S globulin protein in Macadamia integrifolia kernels. Plant J. 19, 699–710. doi: 10.1046/j.1365-313x.1999.00569.x, PMID: 10571855

[B42] Matzke A. J. Matzke M. A. (1998). Position effects and epigenetic silencing of plant transgenes. Curr. Opin. Plant Biol. 1, 142–148. doi: 10.1016/s1369-5266(98)80016-2, PMID: 10066569

[B43] Matzke A. J. Neuhuber F. Park Y. D. Ambros P. F. Matzke M. A. (1994). Homology-dependent gene silencing in transgenic plants: epistatic silencing loci contain multiple copies of methylated transgenes. Mol. Gen. Genet. 244, 219–229. doi: 10.1007/BF00285449, PMID: 8058033

[B44] Moura F. T. Oliveira A. S. Macedo L. L. P. Vianna A. L. B. R. Andrade L. B. S. Martins-Miranda A. S. . (2007). Effects of a chitin-binding vicilin from Enterolobium contortisiliquum seeds on bean bruchid pests (Callosobruchus maculatus and Zabrotes subfasciatus) and phytopathogenic fungi (Fusarium solani and Colletrichum lindemuntianum). J. Agric. Food Chem. 55, 260–266. doi: 10.1021/jf061623k, PMID: 17227051

[B45] Mwangi M. Mwebaze M. Bandyopadhyay R. Aritua V. Eden-Green S. Tushemereirwe W. . (2007). Development of a semiselective medium for isolating Xanthomonas campestris pv. musacearum from insect vectors, infected plant material and soil: Selective medium for Xanthomonas campestris pv. musacearum. Plant Pathol. 56, 383–390. doi: 10.1111/j.1365-3059.2007.01564.x, PMID: 41834780

[B46] Nakato G. V. Christelová P. Were E. Nyine M. Coutinho T. A. Doležel J. . (2019). Sources of resistance in *Musa* to *Xanthomonas campestris* pv. *musacearum*, the causal agent of banana xanthomonas wilt. Plant Pathol. 68, 49–59. doi: 10.1111/ppa.12945, PMID: 41834780

[B47] Namukwaya B. Tripathi L. Tripathi J. N. Arinaitwe G. Mukasa S. B. Tushemereirwe W. K. (2012). Transgenic banana expressing Pflp gene confers enhanced resistance to Xanthomonas wilt disease. Transgenic Res. 21, 855–865. doi: 10.1007/s11248-011-9574-y, PMID: 22101927

[B48] Nayab S. Aslam M. A. ur Rahman S. ud Sindhu Z. D. Sajid S. Zafar N. . (2022). A review of antimicrobial peptides: Its function, mode of action and therapeutic potential. Int. J. Pept. Res. Ther. 28. doi: 10.1007/s10989-021-10325-6, PMID: 41841152

[B49] Ren J. Wen L. Gao X. Jin C. Xue Y. Yao X. (2009). DOG 1.0: Illustrator of protein domain structures. Cell Res. 19, 271–273. doi: 10.1038/cr.2009.6, PMID: 19153597

[B50] ReportLinker . (2022). Global industry and market reports. Available online at: https://www.reportlinker.com/clp/global/3118 (accessed March 19, 2026).

[B51] Ryan E. Galvin K. O’Connor T. P. Maguire A. R. O’Brien N. M. (2007). Phytosterol, squalene, tocopherol content and fatty acid profile of selected seeds, grains, and legumes. Plant Foods Hum. Nutr. 62, 85–91. doi: 10.1007/s11130-007-0046-8, PMID: 17594521

[B52] Sales M. P. Pimenta P. P. Paes N. S. Grossi-de-Sá M. F. Xavier-Filho J. (2001). Vicilins (7S storage globulins) of cowpea (Vigna unguiculata) seeds bind to chitinous structures of the midgut of Callosobruchus maculatus (Coleoptera: Bruchidae) larvae. Braz. J. Med. Biol. Res. 34, 27–34. doi: 10.1590/s0100-879x2001000100003, PMID: 11151025

[B53] Shewry P. R. (1995). Plant storage proteins. Biol. Rev. 70, 375–426. doi: 10.1111/j.1469-185X.1995.tb01195.x, PMID: 7626727

[B54] Šimoníková D. Němečková A. Čížková J. Brown A. Swennen R. Doležel J. . (2020). Chromosome painting in cultivated bananas and their wild relatives (Musa spp.) reveals differences in chromosome structure. Int. J. Mol. Sci. 21, 7915. doi: 10.3390/ijms21217915, PMID: 33114462 PMC7672600

[B55] Stewart C. N. Jr. Via L. E. (1993). A rapid CTAB DNA isolation technique useful for RAPD fingerprinting and other PCR applications. Biotechniques 14, 748–750. 8512694

[B56] Studholme D. J. Wicker E. Abrare S. M. Aspin A. Bogdanove A. Broders K. . (2020). Transfer of Xanthomonas campestris pv. arecae and X. campestris pv. musacearum to X. vasicola (Vauterin) as X. vasicola pv. arecae comb. nov. and X. vasicola pv. musacearum comb. nov. and Description of X. vasicola pv. vasculorum pv. nov. Phytopathology 110, 1153–1160. doi: 10.1094/PHYTO-03-19-0098-LE, PMID: 31922946

[B57] Tam J. P. Wang S. Wong K. H. Tan W. L. (2015). Antimicrobial peptides from plants. Pharm. (Basel) 8, 711–757. doi: 10.3390/ph8040711, PMID: 26580629 PMC4695807

[B58] Tang R. Tan H. Dai Y. Li L. Huang Y. Yao H. . (2023). Application of antimicrobial peptides in plant protection: making use of the overlooked merits. Front. Plant Sci. 14. doi: 10.3389/fpls.2023.1139539, PMID: 37538059 PMC10394246

[B59] Tripathi J. N. Lorenzen J. Bahar O. Ronald P. Tripathi L. (2014a). Transgenic expression of the rice Xa21 pattern-recognition receptor in banana (Musa sp.) confers resistance to Xanthomonas campestris pv. musacearum. Plant Biotechnol. J. 12, 663–673. doi: 10.1111/pbi.12170, PMID: 24612254 PMC4110157

[B60] Tripathi L. Mwaka H. Tripathi J. N. Tushemereirwe W. K. (2010). Expression of sweet pepper Hrap gene in banana enhances resistance to Xanthomonas campestris pv. musacearum: Xanthomonas wilt resistant bananas. Mol. Plant Pathol. 11, 721–731. doi: 10.1111/j.1364-3703.2010.00639.x, PMID: 21029318 PMC6640263

[B61] Tripathi L. Mwangi M. Abele S. Aritua V. Tushemereirwe W. K. Bandyopadhyay R. (2009). Xanthomonas wilt: A threat to banana production in east and central Africa. Plant Dis. 93, 440–451. doi: 10.1094/PDIS-93-5-0440, PMID: 30764143

[B62] Tripathi L. Ntui V. O. Muiruri S. Shah T. Tripathi J. N. (2025). Loss of function of MusaPUB genes in banana can provide enhanced resistance to bacterial wilt disease. Commun. Biol. 8, 708. doi: 10.1038/s42003-025-08093-w, PMID: 40335637 PMC12059061

[B63] Tripathi L. Ntui V. O. Tripathi J. N. (2022). Control of bacterial diseases of banana using CRISPR/Cas-based gene editing. Int. J. Mol. Sci. 23, 3619. doi: 10.3390/ijms23073619, PMID: 35408979 PMC8998688

[B64] Tripathi L. Ntui V. O. Tripathi J. N. (2024). Application of CRISPR/Cas-based gene-editing for developing better banana. Front. Bioeng. Biotechnol. 12. doi: 10.3389/fbioe.2024.1395772, PMID: 39219618 PMC11362101

[B65] Tripathi J. N. Oduor R. O. Tripathi L. (2015). A high-throughput regeneration and transformation platform for production of genetically modified banana. Front. Plant Sci. 6. doi: 10.3389/fpls.2015.01025, PMID: 26635849 PMC4659906

[B66] Tripathi L. Tripathi J. N. Kiggundu A. Korie S. Shotkoski F. Tushemereirwe W. K. (2014b). Field trial of Xanthomonas wilt disease-resistant bananas in East Africa. Nat. Biotechnol. 32, 868–870. doi: 10.1038/nbt.3007, PMID: 25203031

[B67] Tripathi L. Tripathi J. N. Shah T. Muiruri K. S. Katari M. (2019). Molecular Basis of Disease Resistance in Banana Progenitor *Musa balbisiana* against Xanthomonas campestris pv. musacearum. Sci. Rep. 9, 7007. doi: 10.1038/s41598-019-43421-1, PMID: 31065041 PMC6504851

[B68] Vieira Bard G. C. Nascimento V. V. Oliveira A. E. A. Rodrigues R. Da Cunha M. Dias G. B. . (2014). Vicilin-like peptides from Capsicum baccatum L. seeds are α-amylase inhibitors and exhibit antifungal activity against important yeasts in medical mycology: Vicilin-Like Peptides FromCapsicum baccatumL. Seeds. Biopolymers 102, 335–343. doi: 10.1002/bip.22504, PMID: 24817604

[B69] Villao-Uzho L. Chávez-Navarrete T. Pacheco-Coello R. Sánchez-Timm E. Santos-Ordóñez E. (2023). Plant promoters: Their identification, characterization, and role in gene regulation. Genes (Basel) 14, 1226. doi: 10.3390/genes14061226, PMID: 37372407 PMC10298551

[B70] Wang H.-Y. Li P.-F. Wang Y. Chi C.-Y. Jin X.-X. Ding G.-H. (2024). Overexpression of cucumber CYP82D47 enhances resistance to powdery mildew and Fusarium oxysporum f. sp. cucumerinum. Funct. Integr. Genomics 24, 14. doi: 10.1007/s10142-024-01287-1, PMID: 38236308

[B71] Wanjiku M. S. Runo S. Tripathi L. (2021). Genetic transformation of banana with Extracellular Secreted Plant ferredoxin-like protein (ES-Pflp) gene. Asian J. Trop. Biotechnol. 18. doi: 10.13057/biotek/c180202

[B72] Xie Z. Saha N. Chlan C. (2016). Antimicrobial activity of a Cys-rich peptide derived from a Centrosema virginianum vicilin. Am. J. Plant Sci. 7, 11–22. doi: 10.4236/ajps.2016.71011

