# Supplementary material for: Overexpression of MusaVicilin gene for disease resistance in banana
Source: Front Plant Sci. 2026 Mar 23;17:1737976. doi: 10.3389/fpls.2026.1737976 (PMC13051585; doi:10.3389/fpls.2026.1737976)
Supplement: Supplementary file 1 [file Table1.docx]

**Supplementary Tables**

**Supplementary Table 1: Conserved domain identification in eight Vicilin protein sequences**

| **Query ID** | **From** | **To** | **E-Value** | **Bitscore** | **Accession** | **Short name** | **Superfamily** |
| --- | --- | --- | --- | --- | --- | --- | --- |
| CAI8588729.1 | 278 | 383 | 1.93E-59 | 198.51 | cd02245 | cupin_7S_vicilin-like_C | cl40423 |
|  | 51 | 210 | 7.09E-50 | 172.69 | cd02244 | cupin_7S_vicilin-like_N | cl40423 |
| XP_050906640.1 | 282 | 394 | 8.95E-63 | 208.14 | cd02245 | cupin_7S_vicilin-like_C | cl40423 |
|  | 55 | 214 | 6.60E-50 | 173.46 | cd02244 | cupin_7S_vicilin-like_N | cl40423 |
| XP_068467458.1 | 270 | 330 | 9.25E-65 | 211.99 | cd02245 | cupin_7S_vicilin-like_C | cl40423 |
|  | 44 | 206 | 6.82E-52 | 177.7 | cd02244 | cupin_7S_vicilin-like_N | cl40423 |
| ABU45177.1 | 332 | 397 | 3.48E-71 | 230.87 | cd02245 | cupin_7S_vicilin-like_C | cl40423 |
|  | 89 | 280 | 2.43E-53 | 182.71 | cd02244 | cupin_7S_vicilin-like_N | cl40423 |
| ABF94466.1 | 328 | 507 | 1.59E-71 | 228.56 | cd02245 | cupin_7S_vicilin-like_C | cl40423 |
|  | 106 | 271 | 1.27E-49 | 170.77 | cd02244 | cupin_7S_vicilin-like_N | cl40423 |
| XP_065008984.1 | 283 | 328 | 3.63E-75 | 232.41 | cd02245 | cupin_7S_vicilin-like_C | cl40423 |
|  | 60 | 183 | 2.15E-55 | 181.94 | cd02244 | cupin_7S_vicilin-like_N | cl40423 |
| XP_003537573.1 | 247 | 404 | 1.35E-67 | 218.93 | cd02245 | cupin_7S_vicilin-like_C | cl40423 |
|  | 51 | 212 | 1.80E-56 | 190.03 | cd02244 | cupin_7S_vicilin-like_N | cl40423 |
| AAD54245.1 | 462 | 634 | 6.94E-90 | 277.08 | cd02244 | cupin_7S_vicilin-like_N | cl40423 |
|  | 262 | 437 | 4.85E-81 | 253.59 | cd02245 | cupin_7S_vicilin-like_C | cl40423 |

**Supplementary Table 2: List of vicilin protein sequences included and excluded in Phylogenetic analysis**

| **Sequences used in phylogenetic analysis** |
| --- |
|  |
| KAI4314872.1 hypothetical protein L6164_027737 [Bauhinia variegata] |
| MED6155693.1 hypothetical protein PIB30_007201 [Stylosanthes scabra] |
| KAF1861669.1 hypothetical protein Lal_00026080 [Lupinus albus] |
| XP_027362675.1 vicilin-like seed storage protein At2g18540 [Abrus precatorius] |
| XP_019421441.1 PREDICTED: vicilin-like seed storage protein At2g18540 isoform X2 [Lupinus angustifolius] |
| KAK7309281.1 hypothetical protein RJT34_05865 [Clitoria ternatea] |
| KAG5089514.1 hypothetical protein JHK86_002126 [Glycine max] |
| XP_003516577.1 vicilin-like seed storage protein At2g18540 [Glycine max] |
| KAF1873508.1 hypothetical protein Lal_00027546, partial [Lupinus albus] |
| XP_029127602.1 vicilin-like seed storage protein At2g18540 isoform X2 [Cajanus cajan] |
| CAL0331852.1 unnamed protein product [Lupinus luteus] |
| XP_020217979.1 vicilin-like seed storage protein At2g18540 isoform X1 [Cajanus cajan] |
| KAK7406656.1 hypothetical protein VNO78_08285 [Psophocarpus tetragonolobus] |
| KAK7252898.1 hypothetical protein RIF29_37159 [Crotalaria pallida] |
| KAI4327571.1 hypothetical protein L6164_020013 [Bauhinia variegata] |
| KAH1223894.1 Vicilin-like seed storage protein [Glycine max] |
| OIW10993.1 hypothetical protein TanjilG_22800 [Lupinus angustifolius] |
| KAK2372852.1 vicilin seed storage protein [Trifolium repens] |
| KAJ1415290.1 RmlC-like cupin domain superfamily [Sesbania bispinosa] |
| KAK2426690.1 vicilin seed storage protein [Trifolium repens] |
| XP_019444752.1 PREDICTED: vicilin-like seed storage protein At2g18540 [Lupinus angustifolius] |
| TKY70363.1 Vicilin antimicrobial peptides 2-2 [Spatholobus suberectus] |
| CAN8310452.1 unnamed protein product [Astragalus alpinus var. arcticus] |
| XP_057444971.1 vicilin-like seed storage protein At2g18540 [Lotus japonicus] |
| KAF7828828.1 vicilin-like seed storage protein [Senna tora] |
| KAJ1419051.1 RmlC-like cupin domain superfamily [Sesbania bispinosa] |
| PNY13170.1 vicilin-like protein antimicrobial peptides 2-2-like protein, partial [Trifolium pratense] |
| CAL0311508.1 unnamed protein product [Lupinus luteus] |
| RDX62083.1 Vicilin-like seed storage protein [Mucuna pruriens] |
| XP_068467458.1 vicilin-like seed storage protein At2g18540 [Phaseolus vulgaris] |
| KAL2348488.1 hypothetical protein Fmac_002488 [Flemingia macrophylla] |
| AES94919.2 cupin family protein [Medicago truncatula] |
| XP_045818364.1 LOW QUALITY PROTEIN: vicilin-like seed storage protein At2g18540 [Trifolium pratense] |
| XP_054824509.1 vicilin-like seed storage protein At2g18540 [Prosopis cineraria] |
| XP_028775642.1 LOW QUALITY PROTEIN: vicilin-like seed storage protein At2g18540 [Neltuma alba] |
| XP_004512222.4 vicilin-like seed storage protein At4g36700 [Cicer arietinum] |
| CAJ1930033.1 unnamed protein product [Sphenostylis stenocarpa] |
| XP_061342314.1 LOW QUALITY PROTEIN: vicilin-like seed storage protein At4g36700 [Gastrolobium bilobum] |
| KAK7367434.1 hypothetical protein VNO80_09446 [Phaseolus coccineus] |
| XP_054822936.1 vicilin-like seed storage protein At2g18540 [Prosopis cineraria] |
| KAK4262196.1 hypothetical protein QN277_027781 [Acacia crassicarpa] |
| KAI9088918.1 hypothetical protein K1719_029197 [Acacia pycnantha] |
| XP_025698817.1 vicilin-like seed storage protein At4g36700 [Arachis hypogaea] |
| CAI8588729.1 unnamed protein product [Vicia faba] |
| XP_050906640.1 vicilin-like seed storage protein At2g18540 [Lathyrus oleraceus] |
| KAL5099403.1 hypothetical protein RYX36_003730 [Vicia faba] |
| QCD91005.1 reticulocyte-binding protein [Vigna unguiculata] |
| XP_047152775.1 vicilin-like seed storage protein At2g18540, partial [Vigna umbellata] |
| XP_017426216.2 vicilin-like seed storage protein At2g18540 [Vigna angularis] |
| XP_058777883.1 vicilin-like seed storage protein At2g18540, partial [Vicia villosa] |
| GAU16602.1 hypothetical protein TSUD_233540 [Trifolium subterraneum] |
| XP_061342372.1 vicilin-like seed storage protein At2g18540 [Gastrolobium bilobum] |
| XP_028775643.1 vicilin-like seed storage protein At2g18540 [Neltuma alba] |
| WJX74206.1 hypothetical protein P8452_57882 [Trifolium repens] |
| XP_028775644.1 vicilin-like seed storage protein At2g18540 [Neltuma alba] |
| KAI9076134.1 hypothetical protein K1719_041832 [Acacia pycnantha] |
| XP_057457573.1 vicilin-like seed storage protein At2g28490 [Lotus japonicus] |
| KAK7262356.1 hypothetical protein RJT34_29925 [Clitoria ternatea] |
| KAE9620544.1 putative rmlC-like jelly roll protein [Lupinus albus] |
| XP_004487139.1 vicilin-like seed storage protein At2g28490 [Cicer arietinum] |
| KAL9322796.1 hypothetical protein ACSQ67_010849 [Phaseolus vulgaris] |
| RHN75887.1 putative rmlC-like jelly roll protein [Medicago truncatula] |
| XP_028808061.1 vicilin-like seed storage protein At2g28490 [Neltuma alba] |
| XP_027343385.1 LOW QUALITY PROTEIN: vicilin-like seed storage protein At2g28490 [Abrus precatorius] |
| KAK7402142.1 hypothetical protein VNO78_14172 [Psophocarpus tetragonolobus] |
| TKY48497.1 Vicilin antimicrobial peptides 2-2 [Spatholobus suberectus] |
| CAN8323324.1 unnamed protein product [Astragalus alpinus var. arcticus] |
| KAK2380358.1 vicilin seed storage protein [Trifolium repens] |
| XP_027345391.1 vicilin-like seed storage protein At2g28490 [Abrus precatorius] |
| KAI9089097.1 hypothetical protein K1719_029376 [Acacia pycnantha] |
| KAK7404681.1 hypothetical protein VNO78_05637 [Psophocarpus tetragonolobus] |
| MED6168645.1 hypothetical protein PIB30_013374 [Stylosanthes scabra] |
| KAL2328651.1 hypothetical protein Fmac_022078 [Flemingia macrophylla] |
| XP_061359031.1 vicilin-like seed storage protein At2g28490 [Gastrolobium bilobum] |
| XP_019413481.1 PREDICTED: vicilin-like seed storage protein At2g28490 [Lupinus angustifolius] |
| XP_057449027.1 vicilin-like seed storage protein At2g28490 [Lotus japonicus] |
| XP_045828020.1 LOW QUALITY PROTEIN: vicilin-like seed storage protein At2g28490 [Trifolium pratense] |
| XP_004505960.1 vicilin-like seed storage protein At2g28490 [Cicer arietinum] |
| XP_068490201.1 vicilin-like seed storage protein At2g28490 [Phaseolus vulgaris] |
| KAG4978684.1 hypothetical protein JHK86_038158 [Glycine max] |
| KAL4359434.1 hypothetical protein AHAS_Ahas08G0077000 [Arachis hypogaea] |
| CAI8616433.1 unnamed protein product [Vicia faba] |
| XP_003606476.2 vicilin-like seed storage protein At2g28490 [Medicago truncatula] |
| XP_027909974.1 vicilin-like seed storage protein At2g28490 [Vigna unguiculata] |
| KAK7312541.1 hypothetical protein VNO77_36472 [Canavalia gladiata] |
| KAI9089175.1 hypothetical protein K1719_029454 [Acacia pycnantha] |
| XP_054795084.1 LOW QUALITY PROTEIN: vicilin-like seed storage protein At2g28490 [Prosopis cineraria] |
| XP_020214929.1 vicilin-like seed storage protein At2g28490 [Cajanus cajan] |
| ABU45177.1 unknown [Solanum melongena] |
| ABF94466.1 Cupin family protein, expressed [Oryza sativa Japonica Group] |
| XP_065008984.1 vicilin-like seed storage protein At2g18540 [Musa acuminata AAA Group] |
| XP_003537573.1 vicilin-like seed storage protein At2g18540 [Glycine max] |
| KAI5439001.1 hypothetical protein KIW84_024656 [Lathyrus oleraceus] |
| MED6163475.1 hypothetical protein PIB30_080274 [Stylosanthes scabra] |
| KAH1163642.1 hypothetical protein GYH30_001925 [Glycine max] |
| KAG4386526.1 hypothetical protein GLYMA_11G065800v4 [Glycine max] |
| KAK8471773.1 hypothetical protein PHAVU_002G027900 [Phaseolus vulgaris] |
| MCI07167.1 globulin-1 S allele-like, partial [Trifolium medium] |
| KOM45122.1 hypothetical protein LR48_Vigan06g042800 [Vigna angularis] |
| XP_003597174.3 vicilin-like seed storage protein At2g28490 [Medicago truncatula] |
| PNY02685.1 vicilin-like protein antimicrobial peptides 2-2-like protein [Trifolium pratense] |
| XLR64374.1 hypothetical protein S83_015046 [Arachis hypogaea] |
| KAK8463928.1 hypothetical protein PHAVU_011G072800 [Phaseolus vulgaris] |
